# Contriving multi-epitope vaccine ensemble for monkeypox disease using an immunoinformatics approach

**DOI:** 10.3389/fimmu.2022.1004804

**Published:** 2022-10-13

**Authors:** Shahkaar Aziz, Fahad Nasser Almajhdi, Muhammad Waqas, Inam Ullah, Muhammad Adil Salim, Nasir Ali Khan, Amjad Ali

**Affiliations:** ^1^ Institute of Biotechnology and Genetic Engineering, The University of Agriculture, Peshawar, Pakistan; ^2^ Department of Botany and Microbiology, College of Science, King Saud University, Riyadh, Saudi Arabia; ^3^ Department of Biotechnology and genetic Engineering, Hazara University, Mansehra, Pakistan; ^4^ Natural and Medical Sciences Research Center, University of Nizwa, Nizwa, Oman; ^5^ Microbiology Graduate Group, University of California, Davis, Davis, CA, United States; ^6^ Genome Center, University of California, Davis, Davis, CA, United States

**Keywords:** monkeypox, monkeypox virus, multi-epitope vaccine, *in silico* vaccine, immunoinformatics, B-cell epitopes, CTL epitopes

## Abstract

The current global outbreak of monkeypox (MPX) disease, caused by Monkeypox virus (MPXV), has resulted in 16 thousand infection cases, five deaths, and has been declared a global health emergency of international concern by the World Health Organization. Given current challenges in the safety of existing vaccines, a vaccine to prevent MPX infection and/or onset of symptoms would significantly advance disease management. In this context, a multi-epitope-based vaccine could be a well-suited approach. Herein, we searched a publicly accessible database (Virus Pathogen Database and Analysis Resource) for MPXV immune epitopes from various antigens. We prioritized a group of epitopes (10 CD8+ T cells and four B-cell epitopes) using a computer-aided technique based on desirable immunological and physicochemical properties, sequence conservation criteria, and non-human homology. Three multi-epitope vaccines were constructed (MPXV-1–3) by fusing finalized epitopes with the aid of appropriate linkers and adjuvant (beta-defensin 3, 50S ribosomal protein L7/L12, and Heparin-binding hemagglutinin). Codon optimization and *in silico* cloning in the pET28a (+) expression vector ensure the optimal expression of each construct in the *Escherichia Coli* system. Two and three-dimensional structures of the constructed vaccines were predicted and refined. The optimal binding mode of the construct with immune receptors [Toll-like receptors (TLR2, TLR3, and TLR4)] was explored by molecular docking, which revealed high docking energies of MPXV-1–TLR3 (–99.09 kcal/mol), MPXV-2–TLR3 (–98.68 kcal/mol), and MPXV-3–TLR2 (–85.22 kcal/mol). Conformational stability and energetically favourable binding of the vaccine-TLR2/3 complexes were assessed by performing molecular dynamics simulations and free energy calculations (Molecular Mechanics/Generalized Born Surface Area method). *In silico* immune simulation suggested that innate, adaptive, and humoral responses will be elicited upon administration of such potent multi-epitope vaccine constructs. The vaccine constructs are antigenic, non-allergen, non-toxic, soluble, topographically exposed, and possess favourable physicochemical characteristics. These results may help experimental vaccinologists design a potent MPX vaccine.

## Introduction

Monkeypox (MPX) is an emerging zoonotic disease caused by monkeypox virus (MPXV), a member of the *Orthopoxvirus* genus in the family *Poxviridae* (1). The same genus also contains variola (etiological agent of smallpox), vaccinia, and cowpox virus (2). The MPXV is a double-stranded DNA virus with a genome of ~19.7 kb long that codes for 190 open reading frames, which make up most of the material required for viral replication in the cytoplasm of cells (3). MPXV strains are divided into two clades with ~ 0.5% difference in their genomic sequence and circulate in different African regions. The viral isolates of the Central African (Congo Basin) clade are more virulent than the West African clade in humans (4).

The virus was first discovered in monkeys in a Danish laboratory in 1958; thus, it was named ‘monkeypox’. The first human instance was reported in 1970 in the Democratic Republic of the Congo (DRC). Since then, MPX has become endemic in the DRC and has spread to other African nations (5). As of 23rd July 2022, the recent outbreak of MPX has resulted in 16,000 confirmed infection cases and five deaths, as reported to the World Health Organization (WHO) from 75 countries in five of the six WHO regions since 1 January. The global outbreak of MPX now represents a public health emergency of international concern as per the WHO declaration (6). Recent shotgun metagenomics revealed that the MPXV outbreak variant segregates in a significantly divergent phylogenetic branch (~50SNPs), probably signifying a recent evolutionary shift (7).

A diverse group of animal species has been found susceptible to MPXV infection; however, the native host remains unknown. The transmission routes of infection are thought to be saliva/respiratory excretions and contact with lesion exudate or crust material. Another route of viral can be viral shedding *via* faeces (1). Clinical manifestations of MPX are quite like smallpox, but the early lymph node enlargement, often at the onset of fever, distinguishes monkeypox from smallpox. A rash occurs 1–3 days following the onset of fever and lymphadenopathy, with lesions emerging simultaneously and developing at the same rate. Their distribution is mostly peripheral, but it may spread throughout the whole body during a severe illness. Until the lesion desquamates, the infection might linger up to 4 weeks (8). Secondary bacterial infections, respiratory distress, bronchopneumonia, gastrointestinal involvement, dehydration, sepsis, encephalitis, and corneal infection with resulting loss of vision are among the consequences that may occur in patients with MPX (1).

Smallpox vaccination with vaccinia virus (another *orthopoxvirus*) was shown to be nearly 85% protective against MPX in the past (9). Routine smallpox vaccination was terminated after the elimination of smallpox in 1980 (10), and no *orthopoxvirus* vaccination program has been undertaken in almost 40 years. A smallpox vaccine, Dryvax, has also been shown to protect against monkeypox. Nevertheless, the vaccinee and others who come into contact with the vaccinee can suffer from diverse side effects (11). Two vaccines licensed by the U.S. Food and Drug Administration (FDA) for smallpox are available for preventing MPX infection: ACAM2000 and JYNNEOS (also known as Imvamune or Imvanex) (12). The former is a live, replication-competent vaccinia virus (13). Because of this, there is a possibility for adverse side effects (such as progressive vaccinia and eczema vaccinatum) and myopericarditis (estimated incidence of 5.7 per 1,000 primary vaccinees based on clinical trial data); human to human transmission of vaccinia virus can also occur (13, 14). The latter is a replication-deficient modified vaccinia Ankara vaccine recommended for use in preexposure prophylaxis in persons at occupational risk for exposure to orthopoxviruses (13, 15). However, since the mechanism for myopericarditis caused by ACAM2000 is assumed to be immune-mediated, it is unknown if the antigen or antigens that precipitate autoantibodies are also present in JYNNEOS (14). There is no evidence on the efficacy of these vaccinations in the present MPXV outbreak (12).

Heraud et al. revealed protection against MPX by recombinant subunit vaccine (DNA/protein immunization) that provoked helper T-cell (HTL) response and induced the production of limited B-cell epitopes/peptides in the sera of the immunized macaques (11). Franceschi et al. (16) tested a novel vaccination technique on STAT1 mutant mice to examine whether it protects against a fatal MPXV challenge using three recombinant Bovine Herpesvirus 4 (BoHV-4) vectors, each of which expressed a different MPXV glycoprotein, A29L, M1R, or B6R. Their research showed that BoHV-4-based vectors are effective and may be used as a platform for vector-vaccine development. There are, however, no FDA-approved treatments for MPX, and existing vaccinations are restricted owing to safety concerns, and the patients are provided with supportive care and indicative treatment. As a result, new investigations on pathogenesis, prevention, and treatments continue to spark the scientific community’s attention (16). Developing specific therapeutics, including a vaccine against MPX, could help prevent and treat the disease.

Immune responses are crucial to combat viral infection, and a fundamental unit that generates either a cellular or a humoral immune response is called an antigenic epitope. Thus, a multi-epitope vaccine comprising a collection of peptides is a promising strategy to prevent and treat viral infection (17). Compared to experiment-based techniques, developing a multi-epitope-based vaccination is more convenient, cost-effective, and time-saving (18, 19). Additionally, multi-epitope vaccines have the advantage of simultaneously inducing diverse immune responses such as humoral, innate, and cellular responses than monovalent vaccines (20). Multi-epitope-based vaccine models against many viruses, including Lassa, Cytomegalovirus, Ebola, Zika, Chikungunya, Dengue, and Hepatitis C, have been developed utilizing vaccinomics (21). An *in-vitro* study against *Mycobacterium Tuberculosis* verified the viability of the multi-epitope vaccine (21, 22). Moreover, a vaccine developed using a similar technique has been proven to have *in-vivo* prophylactic properties, and many of these vaccines have advanced to the clinical trial stage (23). Therefore, a multi-epitope subunit vaccine may become a viable treatment option for MPXV infection. For that aim, a multi-epitope subunit vaccine for MPXV was designed employing various immunoinformatic methods.

## Materials and methods

### Acquisition and assessment of epitopes

#### Collection of epitopes

**Figure 1:**
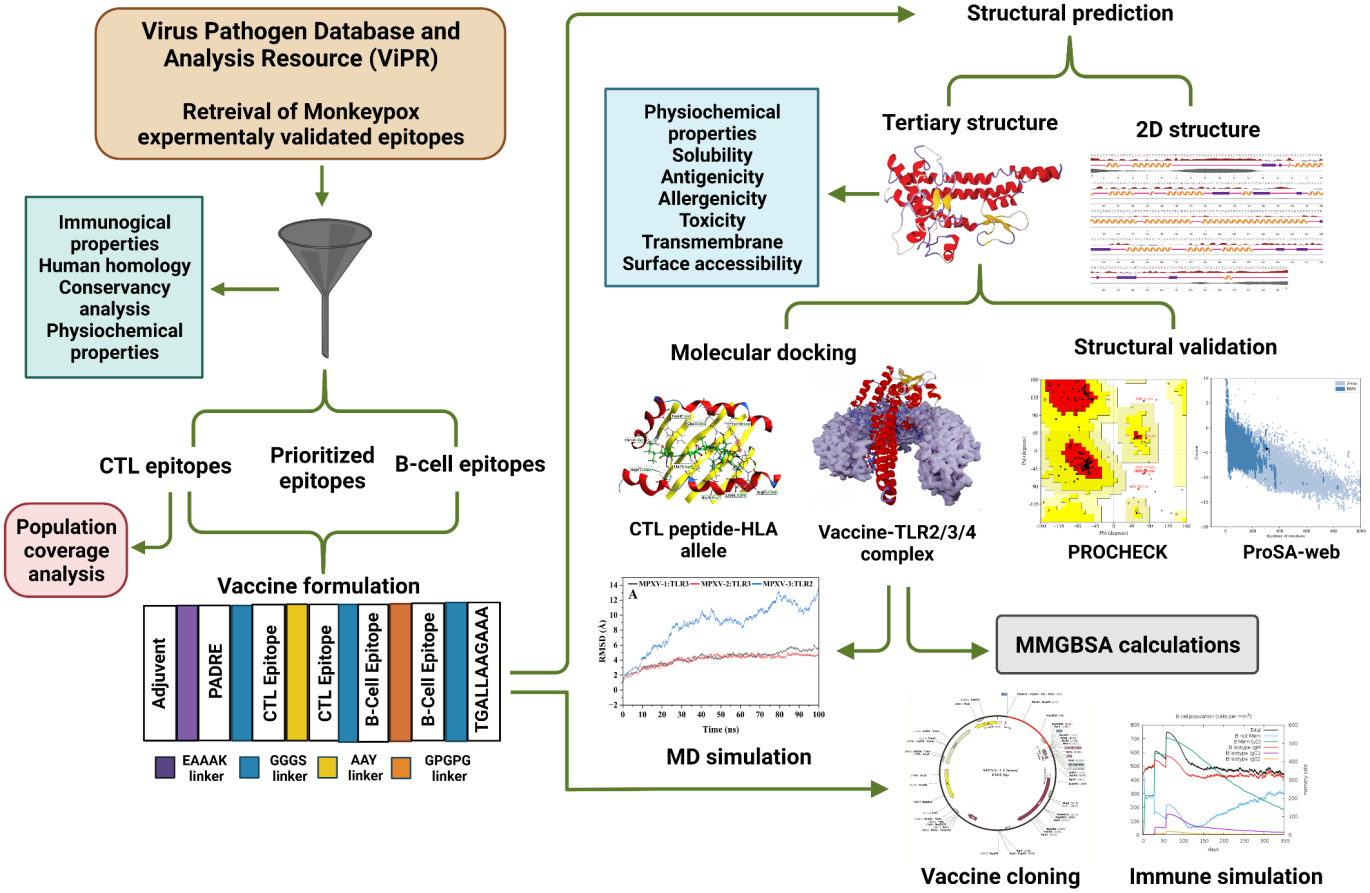
Overall workflow to design vaccine candidates for monkeypox in this study.

The experimentally determined epitopes sequences of the MPXV were obtained from NIAID Virus Pathogen Database and Analysis Resource [(ViPR) (http://www.viprbrc.org); Immune epitopes > Family: *Poxviridae* > Subfamily: *Chordopoxvirinae* > Genus: *Orthopoxvirus* > Species: Monkeypox virus]. Total 139 epitopes sequences, comprising 133 major histocompatibility complex (MHC) class I, one MHC class II, and five B-cell epitopes, were downloaded from ViPR in Excel format. The overall workflow to design a multi-epitope vaccine for MPXV is shown in **Figure 1**.

#### Allergenicity, antigenicity, toxicity and solubility analysis of epitopes

AllerTOP v.2.0 (24) and the Non-Allergen prediction tool of VaxELAN pipeline (25) were utilized to evaluate the allergenicity of the collected epitope. Only epitopes labelled as non-allergen were selected for further analysis. Next, the VexiJen v2.0 server (26) was implemented to test the antigenicity of epitopes with a threshold of 0.5 and the model set as “virus”. Epitope toxicity was checked through ToxinPred server (27), applying the default parameters. Similarly, the virulence potential of the chosen non-allergen, antigenic, and non-toxic epitopes was assessed by using Virulentpred (Cascased SVM module with a threshold of 0.0) (28). Epitopes labelled as “virulent” were selected for the next evaluation. Subsequently, the Innovagen website (http://www.innovagen.com/proteomics-tools) was used to filter non-soluble epitopes.

#### Conservancy, non-homology, and physicochemical property analysis of epitopes

We computed the degree of epitopes conservancy within the MPXV proteins sequences through Immune Epitope Database and Analysis Resource (IEDB) epitope conservancy tool (http://tools.iedb.org/conservancy/). Proteins sequences of Monkeypox virus (NCBI Reference Sequence: NC_063383.1) and Monkeypox virus Zaire-96-I-16 (NCBI Reference Sequence: NC_003310.1) were downloaded in FASTA format from NCBI Reference Sequence Database (https://www.ncbi.nlm.nih.gov/refseq/). The selected epitopes sequences were aligned to their source protein sequences using the IEDB conservancy tool with an identity level set at 100. Epitope sequences were screened against *Homo sapiens* proteome (NCBI taxid: 9606) using the BLASTp tool (29) to discard epitopes that could potentially trigger an autoimmune reaction. Epitopes having percent identity ≤ 70% were retained. Besides, we predicted the similarity of epitopes sequences with the proteome of human gut microbiota with the aid of Non-Bacterial Pathogen prediction tool of VaxELAN pipeline. Physicochemical properties of shortlisted epitopes were estimated *via* the Expasy ProtParam program (https://web.expasy.org/protparam/) as well as Stability and Molecular Weight (kDa) prediction tools of VaxELAN pipeline.

### Proteome screening of monkeypox to predict best-ranking extracellular proteins

Proteins sequences (191) of the reference Monkeypox virus Zaire-96-I-16 (NCBI Reference Sequence: NC_003310.1) were retrieved from NCBI Reference Sequence Database. A state-of-the-art pipeline called VaxELAN was utilized to select the best-ranked outer cellular membrane proteins of MPXV. FASTA sequence of the retrieved proteins was submitted to VaxELAN. Literature-based cut-offs and predefined strategy 1B (integrated with VaxELAN) were applied to find out the eligible protein candidates. Next, the shortlisted proteins’ molecular weight (MW) was computed with the VaxELAN pipeline tool to filter those with MW >110 kDa. To select the final protein candidates, antigenicity and transmembrane (TM) helices of the proteins were checked through VexiJen v2.0 (cut-off score for antigen ≥0.4) and DeepTMHMM (cut-off number of helices ≤ 1) (30), respectively.

### Formulation and evaluation of vaccine constructs

#### Formulation of multi-epitope vaccines

The final epitopes were fused with separate linkers and adjuvant to formulate three multivalent vaccine constructs for MPXV. A potent immunostimulatory adjuvant was added to each multi-epitope vaccine to enhance immunogenicity and trigger long-established innate and adaptive immunity (31). Three different adjuvants were used in the study, including human beta-defensin 3, 50S ribosomal protein L7/L12, and Heparin-binding hemagglutinin [(HBHA) both from *Mycobacterium* sp.],. First, an adjuvant was added to the N-terminal of the vaccine using the EAAAK linker, which is a rigid linker. Following this linker, the pan-HLA DR binding epitopes (PADRE epitope 13aa) sequence was inserted to further increase the vaccine immunogenicity (32). Linkers are essential for achieving extended conformation (flexibility), protein folding, and functional domain separation, all of which contribute to a more stable protein structure (33). The cytotoxic T-lymphocytes [CTLs or major histocompatibility complex (MHC) I binders] epitopes were linked with the AAY linker, and B-cell epitopes were linked with the GPGPG linker. Epitope class was separated with the aid of a GGGS linker (32). Finally, a TAT sequence (11 aa) was adjoined to the vaccine’s C-terminal (34).

#### Population coverage evaluation

The IEDB’s population coverage tool (http://tools.iedb.org/population/) was used to estimate the selected epitopes coverage in the target population. We examined the HLA binding alleles of selected CTL epitopes – predicted by the IEDB SMM method (http://tools.immuneepitope.org/mhci/) with an IC_50_ threshold of 500 – for the population coverage analysis.

#### Prediction of antigenicity, allergenicity, and toxicity of multi-epitope vaccines

Two servers were employed to predict the allergenicity of the modeled vaccines: AllerTOP v.2.0 and AllergenFP v.1.0 (35). Using the VexiJen v2.0 server, the antigenicity of the designed vaccines was evaluated with a threshold value of 0.5. The antigenicity of vaccine constructs was also evaluated using ANTIGENpro, which can be found at http://scratch.proteomics.ics.uci.edu/. ToxinPred server was employed to predict whether the constructed vaccines are non-toxic.

#### Solubility and physicochemical profile evaluation of vaccine constructs

Employing the Protein-Sol (36) and SOLpro (37) servers, the solubility of vaccine constructs was predicted. Protein-Sol server exploits the protein solubility data from an *Escherichia coli* expression system to predict the protein sequence solubility (38). SOLpro implements an SVM-based technique to predict the solubility of protein sequence with an accuracy of 75% (39). Several physicochemical characteristics of vaccine constructs, such as molecular weight, Grand average of hydropathicity (GRAVY), theoretical isoelectric point (pI), instability index, aliphatic index, and half-life, were estimated using the Expasy ProtParam program (https://web.expasy.org/protparam/). Using the DeepTMHMM (30), number of TM helices in the proposed vaccine constructs were predicted. To avoid autoimmunity, the vaccine constructs should not be similar to the human proteins. Therefore, BLASTp tool was used to check the sequence similarity between designed vaccine constructs and human proteins (NCBI taxid: 9606).

### Prediction of secondary structure, solvent accessibility,and disorder regions

With the aid of NetSurfP-3.0 server (https://services.healthtech.dtu.dk/service.php?NetSurfP-3.0), the secondary structure features (α-helixes, β-sheets, and random coils) of vaccine constructs were predicted. This server implements the ESM-1b language model to produce sequence embedding, which is then processed by a deep neural network (40). The solvent accessibility and disorder regions of designed vaccine constructs were also predicted exploiting the NetSurfP-3.0 server.

### Modeling, refinement, and quality validation of tertiary structure

I-TASSER (41) and RoseTTAFold (42) servers were used to predict the tertiary structures of the designed vaccines. Following the primary three-dimensional (3D) modeling, the modeled structures were further improved by employing GalaxyRefine Server (http://galaxy.seoklab.org/). To improve loop or terminal regions in the primary 3D model, this server applies ab initio modeling. Quality validation of the predicted tertiary structure was carried out using the ProSA-web (https://prosa.services.came.sbg.ac.at/prosa.php) and ERRAT (https://saves.mbi.ucla.edu/) server. The Z-Score provided by ProSA-web could be used to assess the overall and local model quality. Besides, the ERRAT quality factor and PROCHECK Ramachandran plot (which showed favourable backbone dihedral angles in relation to amino acid residues in tertiary structure) were further used for model validation (43–45).

### Prediction of confirmational B-cell epitopes

Using the ElliPro web server (http://tools.iedb.org/ellipro/), conformational B-cell epitopes within vaccine constructs were predicted. The default parameters (minimum score of 0.5 and maximum score of 0.6) of this server was kept for epitopes predictions.

### Molecular docking study

#### Peptide modeling and docking with MHC-I molecules

The final CTL epitopes were modeled by PEP-FOLD 3.0 server (46) with 2000 simulations and sOPEP sorting scheme. Molecular docking was performed between CTL epitopes and major histocompatibility class I molecules [HLA-A*0201 (PDB: 4U6X) and HLA-B*15:02 (PDB: 6VB2)] applying the protein-protein (PP) docking protocol of MOE2020 software (47). Before docking, retrieved PDB structures were prepared by removing ligand and water molecules, and energy was minimized with the MOE QuickPrep module. The binding pocket and active site residues of the attached peptide with the PDB structure of selected HLA alleles were selected as the docking site for epitopes. The predominant binding mode (out of five poses) was retained based on strong interaction and high docking score (DS) for each complex. The protein-protein interaction panel of MOE2020 was used for peptide–receptor contact analysis.

#### Molecular docking of vaccine and TLRs

In this study, the binding affinity of vaccine constructs and immune receptors was tested through molecular docking. PP docking protocol of MOE2020 software was applied to perform docking between constructs and several immune receptors, including Toll-like receptors [TLR2 (PDB: 6NIG), TLR3 (PDB: 2A0Z), and TLR4 (PDB: 3FXI)]. All PDB structures were prepared, and energy minimized *via* the MOE QuickPrep module. Attached ligands, heteroatoms, and chains B, C, and D (in the case of TLR2 and TLR4) were removed before docking. Employing a rigid body refinement approach of MOE2020, the final 30 poses were retained during the docking. The PDBsum server [24] was utilized for vaccine–receptor contacts analysis, while structural illustrations were generated using the Blender software (48). Based on a high DS (high negative score), the top-docked vaccine–TLR complex for each construct was further evaluated.

### *In silico* cloning and RNA secondary structure prediction of constructed vaccines

The cDNA of constructed vaccines was obtained by submitting their amino acid sequence into EMBOSS Backtranseq (https://www.ebi.ac.uk/Tools/st/embossbacktranseq/). To optimize the codon usage by *Escherichia coli* (*E. coli*, prokaryotic organism), codon optimization was done *via* Java Codon Adaptation Tool (JCat) (49). The *E. coli* strain K12 was selected to increase vaccine expression efficiency. Additional options, such as avoiding rho-independent transcription termination, prokaryote ribosome attachment site, and the cleavage site of restriction enzymes, were checked for the correct translation of the vaccine gene. Codon optimization index (CAI) and percent GC-content of the output sequence was checked to examine protein’s expression potency. At the N and C-terminal of the vaccine codon sequence, the cleavage site for XhoI and SacI was appended. Using the SnapGene software (https://www.snapgene.com/), the optimized sequence was cloned between XhoI and SacI site in the pET28a (+) expression vector. In addition, minimum free energy mRNA secondary structure from each construct optimized cDNA sequence was predicted using the RNAfold program (50).

### Immune simulation

Computation immune simulation was performed exploiting the C-ImmSim server to ascertain the immune response of the multi-epitope vaccine to the host (51). To describe cellular and humoral profile of mammalian immune system against the formulated vaccine, this server implements the Celada-Seiden model. Following the literature (21), three vaccine doses were injected at suggested 28 days intervals. Total simulation steps were 1050, and time steps of injection were 1, 84, and 170. The rest of the simulation’s parameters were retained as default.

### Molecular dynamics simulations

The vaccine-receptor complex was subjected to molecular dynamics (MD) simulations applying the Particle Mesh Ewald Molecular Dynamics (PMEMD) (52) engine of AMBER22 (53) software. To generate the coordinate and topology file of the system for protein amino acids, the AMBER22 Leap module (53) was employed with ff19SB (54) force field. Monovalent optimal point charge (OPC) ions (55) (Na+ and Cl^–^, ~0.1 M) were introduced to neutralize the system. Solvation of each system was carried out in a truncated octahedral box with an OPC water model and 10% of a buffer. Employing the PMEMD engine (52) on GPUs, parallel scaling in long-range electrostatics was optimized. A two-step initial energy minimization was applied: steepest descent minimization (20000 steps) and conjugate gradient minimization (10000 steps) (56). A Langevin thermostat and an NVE ensemble (microcanonical) were implemented to attain continual heating in 400ps time from 0.1 to 300K before the minimization phase. Besides, a Langevin thermostat (57) was used to adapt the kinetic energy of harmonic oscillators for dynamic propagation with 2.0ps^-1^ collision frequency. The density was adjusted in the 400ps run using the same method as before. Using an NVE ensemble for 400ps, system equilibration was performed at 300K for 2000ps with no restraint and a pressure relaxation time of 2ps. During the equilibration phase, the pressure was upheld by applying the isotropic position scaling method and relaxed for 1ps. SHAKE (58), and particle-mesh Ewald (59) method was used to restrict all hydrogen bonds and estimate long-range electrostatics(threshold of 8Å), respectively. Finally, 100ns production molecular dynamics (MD) was run for each vaccine–TLR complex with same protocol as equilibration phase. For investigating the trajectory, frames were gathered every 10ps.

#### Analysis of simulation trajectories

The resulting trajectory obtained from the MD simulation was investigated using the CPPTRAJ package of AMBER22. Based on cartesian coordinates of C-alpha atoms, overall deviation from the initial structure is measured through the root mean square deviation (RMSD) plot and the resultant root mean square fluctuations (RMSF) (60). The compactness of structure over the simulation duration was examined by the radius of gyration (Rg) and applying the equation mentioned elsewhere (60). Moreover, the protein’s surface properties were analyzed with solvent-accessible surface area (SASA) calculation. Since hydrogen bonds (H-bond) are crucial factors that affect protein stability, we estimated vaccine-receptor interface H-bonds. The H-bond donor and acceptor atom’s distance threshold of 3.5 Å and angel cut-off of 120° were applied in H-bond analysis in this study.

#### Gibbs free energy distribution

The conformational free energy values associated with stable and transient states of the complex were measured hereby. Using the CPPTRAJ package of AMBER22 (60), the system’s Free Energy Landscape (FEL) was investigated. The trajectories data were distributed into 100 bins by principal components, PC1 and PC2, with the highest variations. An artificial barrier was set for bins (having no population) with a population size of 0.5 throughout free energy estimations. Free energy estimations were carried out at 300°C, and all units were measured in kcal/mol.

### Binding free energy calculations

The binding free energy of the vaccine-TLR complex was computed by MM/GBSA (Molecular Mechanics/Generalized Born Surface Area) method (61). The free energy binding of the complex was computed by applying the below formula (62).


ΔGbind = ΔGR+L – (ΔGR + ΔGL)

GR + L, GR, and GL are protein-ligand (vaccine) complex, free protein, and ligand energy, respectively. In addition, applying the equations (63), the ΔG term (free energy) is computed.

## Results

### Collection of monkeypox experimentally validated epitopes from ViPR

A total of 139 experimentally validated epitopes of monkeypox, including 133 MHC class I, one MHC class II, and five B-cell epitopes, were collected from ViPR. These epitopes were then analyzed for several parameters (described in the method section) to select final candidates for vaccine construction.

### Prioritization of monkeypox experimentally validated epitopes

#### Evaluation of immunological properties and solubility of epitopes

Out of 139 epitopes, 46 allergens, 29 non-antigenic, 23 non-virulent epitopes, and 27 poorly soluble predicted by the respective servers were discarded. All epitopes were predicted non-toxic; therefore, no epitope was eliminated after the toxicity analysis. Antigenicity scores of the shortlisted 14 epitopes range from 0.58 to 1.57. While the virulence scores of shortlisted epitopes range from 1.02 to 1.12 (**Table 1**).

**Table 1:** Sequence conservation, human homology, immunological and physicochemical properties of finalized experimentally validated epitopes of MPXV.

Epitopes	AT	Allergenicity	Solubility	Toxicity	Virulence	Conser-vation	Human-homology	II	AI	Half-life(Mammals, hrs)	MW
AIIDIEPDL	1.36	NA	soluble	NT	Virulent (1.06)	100% (2/2)	Non-H	70.73	184.44	4.4	998.14
IHLEWLLGF	1.57	NA	soluble	NT	Virulent (1.06)	100% (2/2)	Non-H	19.61	173.33	20	1127.35
LQKFSFKIA	0.69	NA	soluble	NT	Virulent (1.06)	100% (2/2)	Non-H	18.98	97.78	5.5	1081.32
RTVIHLEWL	1.51	NA	soluble	NT	Virulent (1.06)	100% (2/2)	Non-H	19.61	162.22	1	1166.39
SLKDVLVSV	0.89	NA	soluble	NT	Virulent (1.06)	100% (2/2)	Non-H	–0.54	183.33	1.9	959.15
WKVLSIMAF	0.92	NA	soluble	NT	Virulent (1.09)	100% (2/2)	Non-H	13.17	130	2.8	1094.38
ITVGMLIYSM	0.94	NA	soluble	NT	Virulent (1.06)	100% (2/2)	Non-H	0.51	146	20	1127.42
KLSSYHVVSV	1.00	NA	soluble	NT	Virulent (1.06)	100% (2/2)	Non-H	32.11	126	12.6	1118.30
NVDSTDELM	0.58	NA	soluble	NT	Virulent (1.05)	100% (2/2)	Non-H	13.59	75.56	1.4	1023.08
WAIIPLSASV	1.14	NA	soluble	NT	Virulent (1.06)	100% (2/2)	Non-H	-8.91	166	2.8	1056.27
*LSAATETYSGLT PEQKAYVPAMF	0.74	NA	soluble	NT	Virulent (1.12)	100% (2/2)	Non-H	50.62	63.91	5.5	2475.81
*DSGYHSLDPNAV CETD	0.87	NA	soluble	NT	Virulent (1.02)	100% (2/2)	Non-H	23.82	48.75	1.1	1722.76
*YGAPGSPTNLEFI NTGSSK	1.09	NA	soluble	NT	Virulent (1.07)	100% (2/2)	Non-H	27.09	46.32	2.8	1940.11
*CVRSNEEFDPVDD GPDDETDLSKLSKD	0.61	NA	soluble	NT	Virulent (1.03)	100% (2/2)	Non-H	35.39	50.37	1.2	3026.15

*B-cell epitopes; AT, Antigenicity; NA, Non-allergen; NT, Non-toxic; Non-H, Non-human homologue; II, Instability index; AI, Aliphatic index; MW, Molecular weight.

#### Evaluation of conservancy and human homology of epitopes

All shortlisted 14 epitopes indicated 100% sequence conservation with their source proteins sequences from monkeypox and monkeypox strain Zaire 77-0666. According to the BLASTp homology evaluation, none of the epitopes showed an exact match with *Homo Sapiens* proteins (% identify ≤70%), hinting that the shortlisted epitopes are not expected to trigger autoimmunity.

#### Evaluation of physicochemical properties

The majority of shortlisted peptides have instability index value<40 (stable) and high aliphatic index value (thermostable). The molecular weight of final peptides ranges from 998.14 to 2475.81 Da, and the predicted half-life of the peptides in the mammalian cell is between 1 h to 20 hrs (**Table 1**). The antigen source (or protein name) and entry details of the finalized 14 peptides are provided in **Table S1**.

### Reverse vaccinology pipeline to select exposed proteins

Out of 191 proteins of MPXV, the integrated pipeline finalized eight proteins (A21L, A30L, A43R, B8R, B9R, B20R, C22L, and J2R) as potential vaccine candidates (PVCs) **(** **Figure S1** **).** NCBI IDs, various properties, locus, and proteins names of final vaccine candidates are the given **Table S2**.

### Population coverage analysis

**Figure 2:**
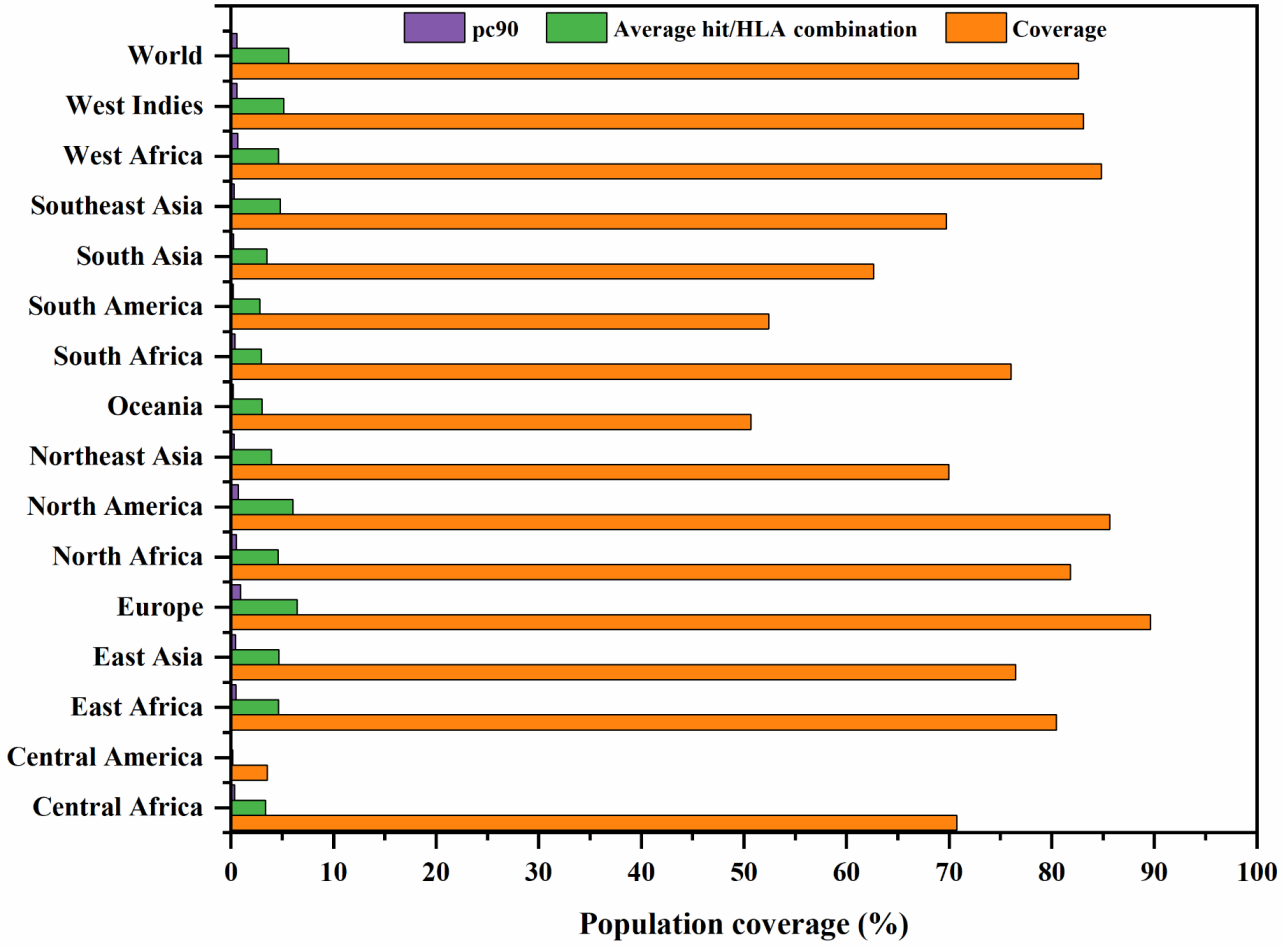
Global population coverage of selected MHC class I epitopes. pc90, the minimum number of epitope hits/HLA combinations recognized by 90% of the population; Coverage, projected population coverage; Average hit/HLA combination, an average number of epitope hits/HLA combinations recognized by the population.

The worldwide population coverage was estimated using the IEDB analysis tool to predict the population coverage of the shortlisted MHC-I epitopes. These epitopes interacted with the assembly of reference alleles **(** **Table S3** **).** The worldwide population coverage of MHC-I epitopes was 82.6%, with a pc90 average of 0.57 and an average hit per HLA antigen of 5.63 (**Figure 2**). The combined population coverage of the selected epitope class was highest in Europe (89.6%), followed by North America (85.63%), West Africa (84.84%), West Indies (83.08%), North Africa (81.83%), and East Africa (80.47%). The selected epitopes had >70% population coverage in East Asia, South Africa, and Central Africa. In addition, with over 60% combined value, final epitopes indicated better coverage in Northeast Asia, Southeast Asia, Southwest Asia, and South Asia populations. In South American and Oceanian populations, population coverage of final epitopes was 52.45% and 50.7%, respectively. On the other hand, in Central America, the population coverage of the shortlisted epitopes class was estimated to be the lowest (3.55%). The average hit per HLA antigen of MHC-I epitopes was 4.14, whereas the pc90 value was calculated to be 0.44. Individual coverage of MHC-I epitopes in the world population is presented in **Table 2**. Among all epitopes, KLSSYHVVSV was predicted to have the highest coverage of 67.23%, followed by ITVGMLIYSM (62.19%), NVDSTDELM (59.24%), LQKFSFKIA (55.78%), and AIIDIEPDL (55.25%). The analysis revealed that selected epitopes could cover a wide range of the human population; thus, they can be promising candidates for formulating polypeptide vaccine.

**Table 2:** Individual world population coverage of selected MHC-I epitopes.

Epitope	Coverage Class I
AIIDIEPDL	55.25%
IHLEWLLGF	19.33%
LQKFSFKIA	55.78%
SLKDVLVSV	49.91%
RTVIHLEWL	49.85%
ITVGMLIYSM	62.19%
NVDSTDELM	59.24%
KLSSYHVVSV	67.23%
WAIIPLSASV	53.43%
WKVLSIMAF	19.33%
Epitope set	82.60%

### Vaccine construction with different adjuvants

The multi-epitope vaccine was constructed by fusing the finalized 10 MHC-I and four B-cell epitopes with adjuvant and linkers. An AAY linker was used to connect MHC-I binding epitopes, which may help create an appropriate site for antigen epitopes to bind to TAP transporters and improve epitope presentation. To join B-cell epitopes, GPGPG linkers were used. This linker stimulates HTL responses while preserving confirmational dependent immunogenicity of T-helpers and antibody epitopes (64). An adjuvant was added to the N-terminal of the construct *via* the EAAAK linker to improve the immunogenicity and immune response against the antigen. A PADRE sequence (13 aa) was also inserted to further increase the vaccine’s immunogenicity. The pan-HLA DR binding epitopes in the vaccine construct enable binding to a wide range of mice and human MHC-II alleles to generate CD4+ helper cell responses (33). A TAT sequence (TGALLAAGAAA 11aa) was added to the C-terminal to allow intracellular delivery of the modeled vaccine (65). Three potential multi-epitope vaccines were constructed. Monkeypox vaccine-1 (MPXV-1) has 306 amino acids (aa) containing beta-defensin 3 adjuvant. MPXV-2 (388 aa) and MPXV-3 (417 aa) have 50S ribosomal protein L7/L12 and HBHA as adjuvants, respectively. The whole aa sequences of modeled vaccines are shown in **Table 3**. The sequence homology of the final vaccine protein to the human protein sequence showed no significant alignments.

**Table 3:** The amino acid sequence of MPXV-1–3 ensemble. An adjuvant is inserted at the N-terminal of the vaccine ensemble. EAAAK linker connecting the PADRE sequence to the adjuvant is represented in bold letters.

Vaccine Name	Adjuvant	Length	Vaccine Ensemble
Monkeypox vaccine- 1 (MPXV-1)	beta-defensin 3	306	GIINTLQKYYCRVRGGRCAVLSCLPKEEQIGKCSTRGRKCCRRKK**EAAAK**AKFVAAWTLKAAA**GGGS**AIIPLSASV**AAY**IHLEWLLGF**AAY**LQKFSFKIA**AAY**RTVIHLEWL**AAY**SLKDVLVSV**AAY**WKVLSIMAF**AAY**ITVGMLIYSM**AAY**KLSSYHVVSV**AAY**NVDSTDELM**AAY**WAIIPLSASV**GGGS**L*SAATETYSGLTPEQKAYVPAMF* **GPGPG** *DSGYHSLDPNAVCETD* **GPGPG** *YGAPGSPTNLEFINTGSSK* **GPGPG** *CVRSNEEFDPVDDGPDDETDLSKLSKD* **GGGS**TGALLAAGAAA
Monkeypox vaccine- 2 (MPXV-2)	50S ribosomal protein L7/L12	388	MAKLSTDELLKEMTLLELSDFVKKFEETFEVTAAAPVAVAAAGAAPAGAAVEAAEEQSEFDVILEAAGDKKIGVIKVVREIVSGLGLKEAKDLVDGAPKPLLEKVAKEAADEAKAKLEAAGATVTVK**EAAAK**AKFVAAWTLKAAA**GGGS**AIIPLSASV**AAY**IHLEWLLGF**AAY**LQKFSFKIA**AAY**RTVIHLEWL**AAY**SLKDVLVSV**AAY**WKVLSIMAF**AAY**ITVGMLIYSM**AAY**KLSSYHVVSV**AAY**NVDSTDELM**AAY**WAIIPLSASV**GGGS** *LSAATETYSGLTPEQKAYVPAMF* **GPGPG** *DSGYHSLDPNAVCETD* **GPGPG** *YGAPGSPTNLEFINTGSSK* **GPGPG** *CVRSNEEFDPVDDGPDDETDLSKLSKD* **GGGS**TGALLAAGAAA
Monkeypox vaccine- 3 (MPXV-3)	Heparin-binding hemagglutinin	417	MAENPNIDDLPLAALGAADLALATVNDLIANLRERAEETRAETRTRVEERRARLTKFQEDLPEQFIELRDKFTTEELRKAAEGYLEAATNRYNELVERGEAALQRLRSQTAFEDASARAEGYVDQAVELTQEALGTVASQTRAVGERAAKLVGIEL**EAAAK**AKFVAAWTLKAAA**GGGS**AIIPLSASV**AAY**IHLEWLLGF**AAY**LQKFSFKIA**AAY**RTVIHLEWL**AAY**SLKDVLVSV**AAY**WKVLSIMAF**AAY**ITVGMLIYSM**AAY**KLSSYHVVSV**AAY**NVDSTDELM**AAY**WAIIPLSASV**GGGS** *LSAATETYSGLTPEQKAYVPAMF* **GPGPG** *DSGYHSLDPNAVCETD* **GPGPG** *YGAPGSPTNLEFINTGSSK* **GPGPG** *CVRSNEEFDPVDDGPDDETDLSKLSKD* **GGGS**TGALLAAGAAA

Different linkers (GGGS, AAY, and GPGPG) are also represented in bold letters. B-cell epitopes are italicized. A TAT sequence (TGALLAAGAAA) is inserted at the N-terminal of the vaccine ensemble.

### Assessment of antigenicity, allergenicity, and toxicity of constructed vaccines

VexiJen v2.0 server predicted the constructed vaccines as potential antigens with antigenicity scores of 0.63 (MPXV-1), 0.55 (MPXV-2), and 0.59 (MPXV-3). ANTIGENpro server also confirmed the probable antigen character of the proteins with a prediction score >0.7. AllerTOP v.2.0 and AllergenFP v.1.0 labelled the potential vaccine constructs as non-allergen. Similarly, the designed constructs were predicted non-toxic by the ToxinPred server (**Table 4**).

**Table 4:** Evaluation of Estimated solubility, immunological, and physicochemical properties of designed vaccine constructs by Expasy ProtParam server.

**Property**	**MPXV-1**	**MPXV-2**	**MPXV-3**
**SOLpro**	0.76	0.96	0.93
**Protein-Sol**	0.46	0.65	0.52
**Molecular weight**	31984.51 Da	39930.45 Da	44170.60 Da
**Formula**	C_1435_H_2228_N_374_O_428_S_13_	C_1804_H_2823_N_445_O_556_S_9_	C_1964_H_3071_N_523_O_619_S_8_
**Theoretical pI**	7.66	4.68	4.70
**Ext. coefficient**	53, 330 M^-1^ cm^-1^	49, 975 M^-1^ cm^-1^	54, 445 M^-1^ cm^-1^
**Instability index**	23.82 (stable)	19.39 (stable)	27.43 (stable)
**Aliphatic index**	84.02 (thermostable)	92.99 (thermostable)	87.96 (thermostable)
**Half-Life**	30 hrs (mammalian reticulocytes, *in-vitro*). >20 hrs (yeast, *in-vivo* >10 hrs (*E. coli*, *in-vivo*).	30 hrs (mammalian reticulocytes, *in-vitro*). >20 hrs (yeast, *in-vivo*). >10 hrs (*E. coli*, *in-vivo*).	30 hrs (Mammalian reticulocytes, *in-vitro*). >20 hrs (yeast, *in-vivo*). >10 hrs (*E. coli*, *in-vivo*).
**Allergenicity**	AllerTOP v.2.0 (Non-allergen), AllergenFP v.1.0 (Non-allergen)	AllerTOP v.2.0 (Non-allergen), AllergenFP v.1.0 (Non-allergen)	AllerTOP v.2.0 (Non-allergen), AllergenFP v.1.0 (Non-allergen)
**Antigenicity**	ANTIGENpro (0.73), VexiJen v2.0 (0.63)	ANTIGENpro (0.75), VexiJen v2.0 (0.55)	ANTIGENpro (0.72), VexiJen v2.0 (0.59)
**Toxicity**	Non-toxic	Non-toxic	Non-toxic
**TM helices**	None	1	1

### Analysis of solubility and physicochemical properties of the designed vaccine constructs

Protein-Sol and SOLpro servers predicted the constructed vaccines as soluble. MPXV-1–3 vaccine has MW of 31.98kDa, 39.93kDa, and 44.17kDa and theoretical pI of 7.66 (alkaline), 4.68 (acidic), and 4.70 (acidic), respectively. The estimated MW of constructs is suitable for vaccine application, and they could be purified easily (>110kDa). The computed instability index of MPXV-1 (23.82), MPXV-2 (19.39), and MPXV-3 (27.43) indicated that the designed vaccine constructs are stable proteins (value >40 indicates a stable vaccine). The aliphatic index of the proteins ranges from 84.02 to 92.99, classifying the formulated vaccine constructs as highly thermostable. The estimated half-life of all constructs is 30 hrs *in-vitro.* The estimated half-life of all constructs in yeast and *E. coli* (*in-vivo*) is more than 20 hrs and 10 hrs, respectively. The predicted TM helices (0–1) also suggest the suitability of vaccines for application (**Table 4**).

### Prediction of two-dimensional structure, solvent accessibility, and disorder regions

**Figure 3:**
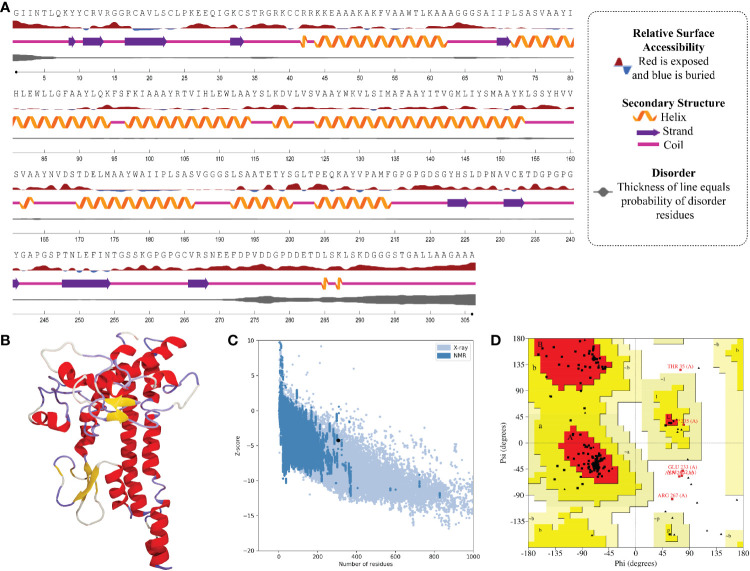
Structural analysis and validation of MPXV-1 **(A)** The predicted secondary structure features, relative surface accessibility, and disorder regions using NetsrurfP-3.0 server **(B)** Final 3D modeled structure of the constructed vaccine **(C)** Z-score graph *via* ProSA-web showing the modeled 3D structure corresponds to X-Ray crystallographic determined structure for the protein of similar sizes **(D)** Ramachandran plot details of the final modeled structure of designed vaccine.

**Figure 4:**
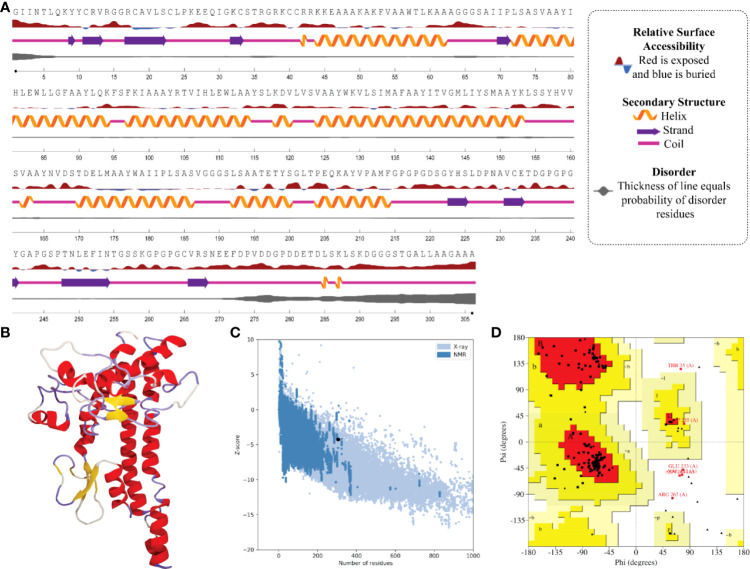
Structural analysis and validation of MPXV-2 **(A)** The predicted secondary structure features, relative surface accessibility, and disorder regions using NetsrurfP-3.0 server **(B)** Final 3D modeled structure of the constructed vaccine **(C)** Z-score graph *via* ProSA-web showing the modeled 3D structure corresponds to X-Ray crystallographic determined structure for the protein of similar sizes **(D)** Ramachandran plot details of the final modeled structure of designed vaccine.

**Figure 5:**
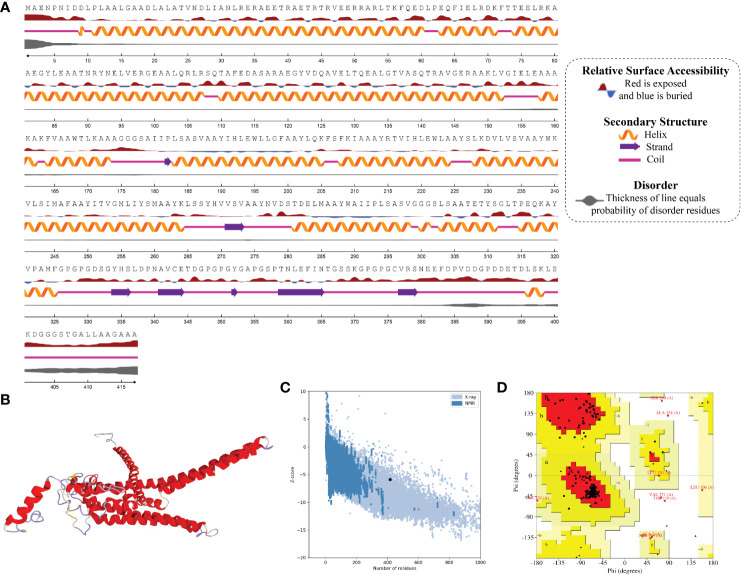
Structural analysis and validation of MPXV-3 **(A)** The predicted secondary structure features, relative surface accessibility, and disorder regions using NetsrurfP-3.0 server **(B)** Final 3D modeled structure of the constructed vaccine **(C)** Z-score graph *via* ProSA-web showing the modeled 3D structure corresponds to X-Ray crystallographic determined structure for the protein of similar sizes **(D)** Ramachandran plot details of the final modeled structure of designed vaccine.

In the result of the MPXV-1 secondary structure, there were 40.19% β-sheets, 11.03% α-helix, and 48.37% random coil. The secondary structure of MPXV-2 and 3 revealed 44.84% and 61.63% β-sheets, 13.14% and 5.27% α-helix, and 43.29% and 33.33% of the random coil, respectively. The solvent accessibility of three vaccine constructs showed sufficient exposure of selected epitopes to solvent; however, few MHC-I epitopes (AIIPLSASV, IHLEWLLGF, and WAIIPLSASV) were moderately exposed in all designed vaccines. In the results of disorder regions prediction, starting and ending residues of the three vaccine constructs were predicted disordered. There were 91.51% ordered and 8.49% disordered residues in the MPXV-1, 86.35% ordered and 13.65% disordered residues in MPXV-2, and 94.73% ordered and 5.27% disordered residues in the MPXV-3 structure. Secondary structures, solvent accessibility, and disorder regions predictions of the three vaccine constructs are illustrated in **Figures 3**–**5**.

### Prediction, optimization, and quality assurance of tertiary structure

The tertiary structure of MPXV-1–3 provided by I-TASSER was predicted to have better structural quality and was refined by the GlaxayRefine server, which yielded five optimized models. The details of five generated models for each construct are given in **Table S4**. Model 1 of MPXV-1 and model 5 of MPXV-2 and 3 had top Ramachandran favored, thus selected for docking study. A model with more residues located in the favored region of the Ramachandran plot and fewer residues in the additionally allowed, generously allowed, and disallowed region was considered ideal. The initial model (I-TASSER) and refined model (GalaxyRefine) were compared for Ramachandran plot details *via* PROCHECK.

The initial model of MPXV-1 had 89.5% residues in the most favoured regions of the Ramachandran plot, 7.4% in additional allowed regions, 1.2% in generously allowed regions, and 2.0% in the disallowed region. The refined model of MPXV-1 had 93.0% residues in most favoured regions, 4.7% in additional allowed regions, 1.2% in generously allowed regions, and 2.0% in the disallowed regions. Similarly, the initial MPXV-2 structural model had 88.2%, 6.9%, 2.1%, and 2.7% residues in the Ramachandran favoured, additionally allowed, generously allowed, and disallowed regions, respectively. On the other hand, the improved model had 89.4%, 6.0%, 1.5%, and 3.0% residues in the Ramachandran favoured, additionally allowed, generously allowed, and disallowed regions, respectively. For the MPXV-3 refined model, the Ramachandran plot distribution of residues was as follows: 94.8% in most favored regions (initial model 92%), 2.8% in additional allowed regions (initial model 5.8%), 1.1% in generously allowed regions (initial model 1.4%), 1.1% in disallowed regions (same as the initial model).

ProSA-web and ERRAT were employed to verify the quality and potential errors in the three-dimensional (3D) model of the final vaccine. The model with a lower Z-score was considered a high-quality one. The Z-score of the refined model of MPXV-1–3 was –4.25, –5.62, and –5.87, respectively. ProSA-web Z-score and Ramachandran plot details of MPXV-1–3 are provided in **Figures 3** **–** **5** and **Table 5**. A higher value of the overall quality factor provided by ERRAT indicates a high-resolution structure for the given protein. The refined vaccine models showed a quality factor score >92%, suggesting the final models were of higher quality **(** **Figures 3** **–** **5** **).**

**Table 5:** Validation of the modeled 3D structures of constructed vaccines.

Vaccine	ProSA-web		Ramachandran Plot details								ERRAT score	
	Z-score		Most favoured regions		Additional allowed regions		Generously allowed regions		Disallowed regions			
	Before	After	Before	After	Before	After	Before	After	Before	After	Before	After
**MPXV-1**	–4.05	–4.25	89.5%	93.0%	7.4%	4.7	1.2%	0.4%	2.0%	2.0%	96.64	94.17
**MPXV-2**	–5.35	–5.62	88.2%	89.4%	6.9%	6.0%	2.1%	1.5%	2.7%	3.0%	97.34	96.81
**MPXV-3**	–5.72	–5.87	92.0%	94.8%	5.8%	2.8%	1.1%	1.4%	1.1%	1.1%	98.28	92.38

### Prediction of discontinuous B-cell epitopes

Tertiary structure and folding of the new protein (vaccine construct 3D model) can result in a new discontinuous B-cell epitope, prompting further predictions. Therefore, such epitopes in the refined tertiary structure of each construct were predicted and enlisted in **Figure S2-S4**. Briefly, seven discontinuous B-cell epitopes were predicted for MPXV-1 involving a total of 171 residues with a score ranging from 0.55 to 0.76. For MPXV-2, four discontinuous B-cell epitopes were predicted, which involved a total of 207 residues with a score in the range of 0.62 to 0.86. In addition, predicted discontinuous B-cell epitopes for MPXV-3 (score ranged from 0.52 to 0.90) covered 221 residues.

### Molecular docking analysis

Finalized CTL peptides were modeled and subjected to molecular docking with their common MHC-I allele (HLA-A*0201 and HLA-B*15:02). All 10 peptides showed stable and high-affinity binding with the HLA-A*0201 molecule, as shown by their high DS ranging from –9.43 to –11.13 kcal/mol. With HLA-A*0201 molecule, RTVIHLEWL peptide showed the highest DS (–11.13 kcal/mol) and several H-bonds, followed by ITVGMLIYSM (–10.7761 kcal/mol) and IHLEWLLGF (–10.6489 kcal/mol). Likewise, the selected CTL peptides indicated a substantial affinity for the HLA-B*15:02 molecule (DS in the range of –9.53 to –12.55 kcal/mol). Peptide WKVLSIMAF also exhibited the highest DS of –12.55 kcal/mol with HLA-B*15:02 molecule, followed by LQKFSFKIA and RTVIHLEWL (DS: –11.84 kcal/mol for each epitope). Peptide–MHC-I molecule docking calculations, interface interactions, and predominant binding mode with the respective HLA receptor are provided in the **Tables S5, S6** and **Figures S5, S6**.

Antigen molecule-immune receptor interactions are essential for transporting antigenic molecules and prompting the immune response pathway. To study the strength of binding energy and molecular interaction between various immune receptors (TLR2, TLR3 and TLR4) and modeled vaccines (MPXV-1–3), molecular docking was performed. The optimal-docked complex was selected out of 30 poses, indicating vaccine constructs’ efficient binding at the receptor-binding domain. Amongst all TLR receptors, TLR3 exhibited high-affinity binding with MPXV-1 (–99.09 kcal/mol) and MPXV2 (–98.68 kcal/mol). In contrast, TLR2 showed the best docking energy with MPXV-3 (–85.22 kcal/mol) among all TLR receptors. Thus, molecular docking analysis implies successful binding and substantial affinity between immune receptors and proposed vaccine constructs. Vaccine-TLRs docking estimation results are supplied in the **Table 6**.

**Table 6:** Molecular docking results of MPXV-1–3 with selected TLRs.

Vaccine	Receptor PDB ID	Name	DS (kcal/mol)	E-Confirm	E-Refine
MPXV-1	6NIG	TLR2	–77.56	–3576.82	–77.56
MPXV-1	2A0Z	TLR3	**–99.09**	–3610.45	–99.09
MPXV-1	4G8A	TLR4	–78.11	–3598.26	–92.27
MPXV-2	6NIG	TLR2	–91.67	–8074.44	–98.68
MPXV-2	2A0Z	TLR3	**–98.68**	–8081.54	–47.13
MPXV-2	4G8A	TLR4	–94.23	–8070.04	–94.23
MPXV-3	6NIG	TLR2	**–85.22**	–12495.07	–85.22
MPXV-3	2A0Z	TLR3	–80.24	–12496.43	–80.24
MPXV-3	4G8A	TLR4	–80.91	–12508.11	–80.91

E-Conf, Energy of Confirmation: E-Refine, Energy of refinement; DS, Docking score.

**Figure 6:**
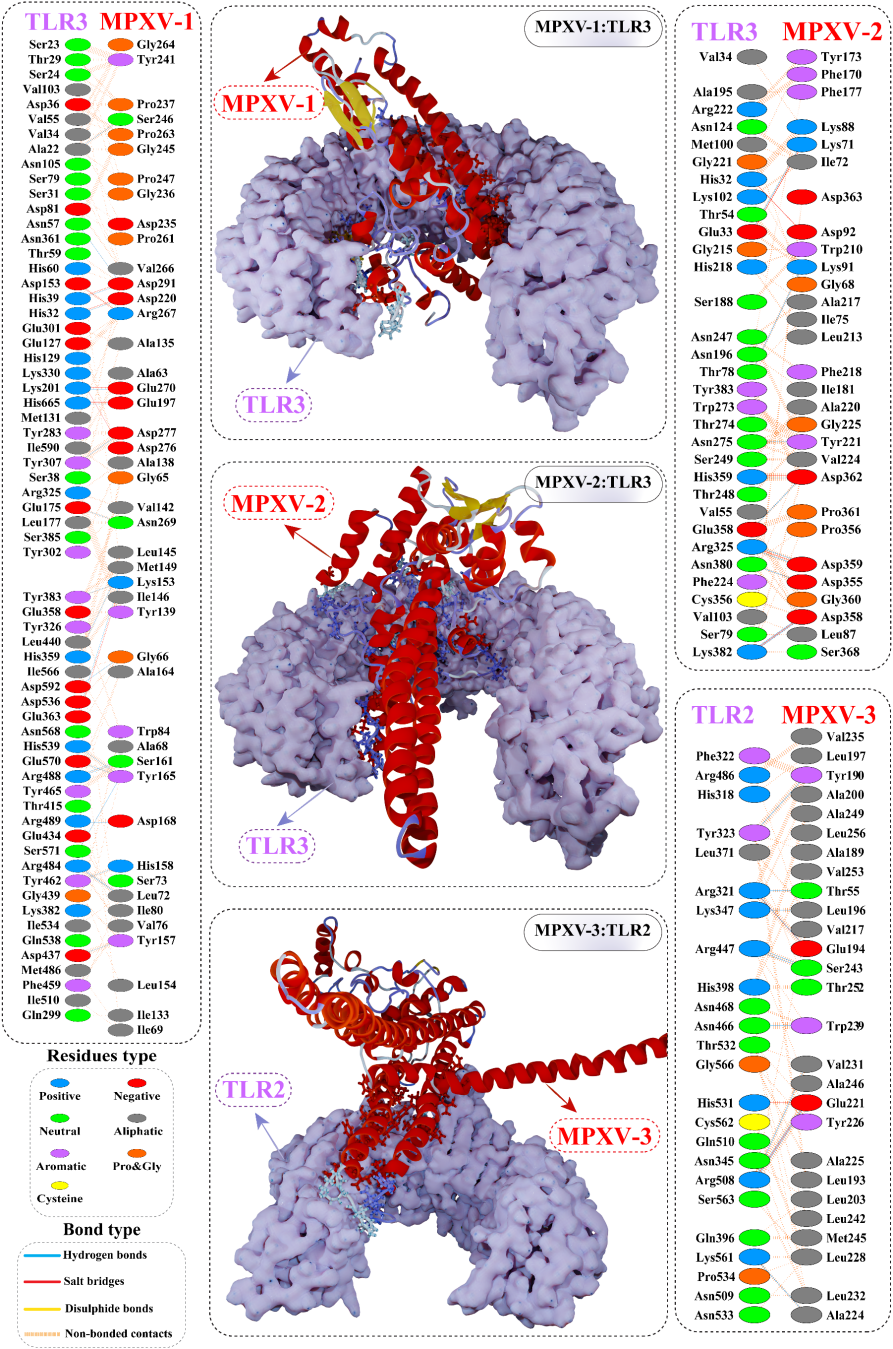
Optimal binding mode of MPXV vaccine construct with human Toll-like receptor-3 (TLR3) and TLR2 receptor. The docked complexes are illustrated in the middle. Protein-protein contacts of the vaccine construct with TLR3/2 receptor are shown on either side. Hydrogen bonds are depicted in blue lines. Residue type is shown with a distinctive colour.

Interface contact analysis of MPXV-1 and 2 with TLR3 receptor and MPXV-3 with TLR2 receptor is discussed herein due to their substantial docking energy. Strong molecular interactions were noticed for these complexes, including hydrogen bonds (H-bonds), salt bridges, and non-bonded contacts. With a bond distance< 2.75Å, MPXV-1 residues that made H-bond with TLR3 are: Met149, Lys153, Tyr157, Ser161, Ala164, Tyr165, Asp220, Ser246, Asp276, Asp277, Asp280, Asp291. MPXV-1 residues that mediated H-bond with a bond distance< 3Å included Gly66, Leu72(2x), Leu145, Lys153, Tyr165(2x), Asn166, Gly240, Tyr241, Pro244, Gly245, Asn269, Glu270, and Asp291(2x). Moreover, His158 and Ala164 of MPXV-1 formed H-bond with TLR3 with a bond distance >3Å. Furthermore, salt bridge interaction was shown by MPXV-1 residues [Asp220, Arg267, Glu270, Asp280, and Asp280 (2x)] within a 3Å bond distance with TLR3. The detailed atom-atom contacts between MPXV-1 and TLR3 interface are given in the **Figure 6** and **Table S7**.

In the case of MPXV-2, the following residues of the modeled vaccine mediated H-bond with TLR3 having a bond distance< 2.75Å: Lys3(2x), Glu30, Glu279, Glu287, Gln288, Asp317 (2x), Ser340 (2x), Glu352, Asp362, and Ser371. Similarly, MPXV-2 residues, such as Ala33, Ala145, Gly147, Glu315 (2x), Asp317, Asn336, Lys341, Glu352, Asp362, and Lys372 formed H-bond with TLR3 with bond distance in a range of 2.76 to 3Å. Residues, Ala33, Gly283, Lys341, and Ser340 also made H-bond with TLR3 with a bond distance >3Å. In addition, several residues of the construct showed salt bridge interactions [Glu279, Glu287 (2x), Asp317 (2x), and Lys341], with TLR3 having a bond distance ranging from 2.72Å to 3.66Å. The detailed atom-atom contacts between MPXV-2 and TLR3 interface are given in **Figure 6** and **Table S8**.

In the case of MPXV-3, residues Tyr190, Glu194, and Glu221 (2x) mediated H-bond with TLR2 with >2.75Å bond distance. Likewise, the construct residues that showed hydrogen bonding with TLR2 with a bond distance in a range of 2.76Å to 2.97Å are Thr55, Gln58, Leu193, Phe199, Glu194, Val217, His219, Leu220(2x), Glu221 (2x), Ala225, Lys229 (2x), Val231, Ser243(2x), and Ala246. Moreover, Glu194 and Glu221(3x) residues of MPXV-3 interacted through salt bridges with TLR3 within a 2.81Å bond distance. The detailed atom-atom contacts between MPXV-3 and TLR2 interface are given in **Figure 6** and **Table S9**.

### Molecular dynamics simulation

**Figure 7:**
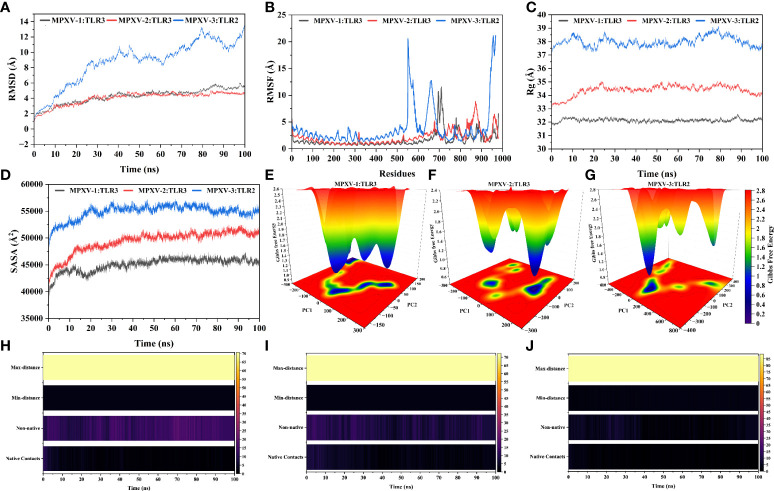
Molecular dynamics simulation of MPXV vaccines and TLR3/2 complex at 100ns **(A)** Root means square deviation (RMSD) plot of the complex, demonstrating slight fluctuations **(B)** Root means square fluctuation (RMSF) plot of the docked complex. TLR3 receptor starts at residue number 1 and goes to residue number 675. MPXV-1 starts at residue number 676 and goes to residue number 982. MPXV-2 starts at residue number 676 and goes to residue number 1064. TLR2 receptor starts at residue number 1 and goes to residue number 550. MPXV-3 start at residue number 551 and residue number 968 **(C)** Radius of Gyration plot of the docked complex **(D)** Variations in Solvent-Accessible Surface Area (SASA) profile of the docked complex during the simulation. Free energy landscapes (FELs) of the **(E)** MPXV-1–TLR3 **(F)** MPXV-2–TLR3 **(G)** MPXV-3–TLR2 docked complex. In the graph, red, yellow/green, and light-to-dark blue depicts high, intermediate, and low/stable energy states, respectively. Native and non-native contacts between **(H)** MPXV-1–TLR3 **(I)** MPXV-2–TLR3 **(J)** MPXV-3–TLR2 interface during 100ns.

The predominant binding mode of the vaccine-TLRs complex was evaluated for structural stability by 100ns MD simulation. RMSD versus time plot was used to characterize the conformational stability of the complexes (**Figure 7A**). The mean RMSD of 4.49Å, 4.37Å, and 9.33Å was obtained for MPXV-1–TLR3, MPXV-2–TLR3, and MPXV-3–TLR2 complex, respectively. The first two complexes showed stable behaviour throughout the simulation. However, in the case of the MPXV-3–TLR2 complex, a sharp increase in RMSD (from 0.55Å to 9.34Å) was noticed till 40ns. Then the system underwent fluctuations in RMSD and remained unstable till the end. The residue flexibility of the vaccine-TLRs complex was evaluated through RMSF to understand the mobility of contact residues and their influence on the binding of the construct with the receptor (**Figure 7B**). The average RMSF of 1.15Å, 1.74Å, and 2.63Å was detected for the MPXV-1, MPXV-2, and MPXV-3 complex, respectively. In the case of the MPXV-1–TLR3 complex, a maximum RMSF of 11.57Å was noticed for Thr35 (MPXV-1; adjuvant) and a minimum of 0.57Å for Cys335 (TLR3). Besides, interacting residues of the MPXV-1 construct (H-bonded residues stated in docking results) with the receptor showed stable RSMF, estimated< 3Å. For the MPXV-2–TLR3 complex, the highly mobile residue was Ala196 (RSMF =8.63Å, MPXV-2 epitope) and the lowest mobile residue was Asn231 (0.60Å, TLR3). In addition, most of the MPXV-2 interacting residues (H-bonded residues stated in docking results) had RMSF ≤ 4.6Å. The highest and least fluctuating residue in the case of MPXV-3–TLR2 complex was Gly405 (RMSF = 20.82Å, MPXV-3 linker) and Ser342 (RMSF=1.3381, TLR3), respectively. The estimated RMSF for interacting residues of MPXV-3 (H-bonded residues stated in docking results) with receptor was ≤ 3.8Å. In the MPXV1/3 system, adjuvant residues of the construct fluctuated significantly. In contrast, in the MPXV-2 system, residues comprising a few MHC-I epitopes and AAY linkers revealed higher flexibility. These motion variations over the simulation run seem important for the vaccine construct to attain a stable confirmation for appropriate exposure to the host immune system.

The Rg profile of vaccine-TLRs complexes was plotted, as shown in **Figure 7C**, to examine the structural compactness and regular packing of secondary structural elements in the 3D structure of the complex. Less stable folding of secondary structural elements results in loose packing of the structure, which is indicated by a high Rg descriptor. The mean Rg value of 32.12Å and 34.45Å and 37.94Å was estimated for MPXV-1–3 bound complexes, respectively. Consistent with the RMSD results, the Rg plot of these complexes stayed stable during the simulation (although the MPXV-3 system experienced fluctuations), which points to fine compactness of the tertiary structure. Alteration in the complex volume was studied by the SASA analysis (**Figure 7D**), which yielded a mean SASA of 45382.27Å, 50046.83 Å, and 55170.52Å for MPXV-1–3 systems, respectively. The SASA values of each complex gradually increased till 30ns and then sustained around the mean SASA value till the end of the simulation, indicating the expansion in the protein complex volume during the initial phase.

The transition phase of each MPXV–TLR complex was explored through the free energy landscape (FEL). To investigate the progression from initial to metastable state, the first two eigenvectors from the resulting trajectory were plotted to generate FEL (**Figures 7E-G**). In addition, the low energy state of each complex system was displayed to analyze structural alteration after the attachment of the construct with the receptor. Across the simulation, high translation confirmation was seen within each system. Allocation of various metastable states (high energy level red, intermediate energy level yellow, stable energy state blue) reveal structural adjustment during the simulation.

The patterns of H-bonds for the three complexes were estimated in each frame within 3Å to probe the strength of intermolecular associations across the simulation period. For the MPXV-1–TLR3 complex, H-bond between Leu145_MPXV-1_ and His338_TLR3_, Tyr165 and Glu549, and Glu270 and Lys180 had a significant retention time of 52%, 33%, and 26% during the simulation, indicating these interactions are crucial for the specificity and strength of intermolecular association. Besides, Lys153, Asp280, and Tyr157 of the vaccine mediated H-bond with the receptor’s Glu342, Arg310, and Asp416, respectively, with over 10% occupancy. All H-bond occupancy details between the MPXV-1–TLR3 interface are provided in **Table S10**. In the case of the MPXV-2–TLR3 complex, a high-frequency H-bond was detected between Gly315_MPXV-2_ and Lys180_TLR3_ (75%), Lys341 and Asp259 (56%), and Gly317 and Arg230 (~54%), Thr31 and Arg201 (42%), and Asp317 and Lys179 (36%) throughout the simulation. Additionally, Thr28, Ser343, Gly147, and Gln288 residues of the vaccine construct maintained H-bond with the receptor’s Arg230, Glu285, Glu413, and Ser17 residues for the considerable period of simulation (>10% occupancy). The detailed H-bond interactions with percent occupancy between MPXV-2–TLR3 interface are enlisted in **Table S11**. Similarly, H-bond between Glu221_MPXV-3_ and Arg482_TLR2_ [(2x), 35% occupancy], Ser243_MPXV-3_ and Arg421_TLR2_(23%), and Val217_MPXV-3_ and Arg 482_TLR2_ (18%) contributed significantly towards the vaccine-receptor binding strength. The detailed H-bonds for MPXV-3–TLR2 complex are given in **Table S12**. Furthermore, changes in native contacts between vaccine-TLRs complex with simulation time can be seen in **Figures 7H-J**, whereby non-native contacts interactions between vaccine and TLRs receptors were improved after simulation, suggesting the complexes’ stability.

### Codon optimization

After reverse translation, adaptability, and preference analysis of codon, the DNA sequence of MPXV-1–3 constructs were 915, 1164, and 1248 nucleotides in length. Furthermore, the codon adaptation index (CAI) of MPXV-1(1), MPXV-2 (1), and MPXV-3 (0.98) were greater than 0.8, meaning that the optimized codon has high adaptability for *E. coli* usage. In addition, the GC-content of the optimized codon was 52.45%, 51.37%, and 53.60% for MPXV-1–3, respectively, implying the stable and high expression potential of the designed constructs in the *E. coli* (strain K12) system.

### *In silico* cloning and prediction of RNA secondary structure

**Figure 8:**
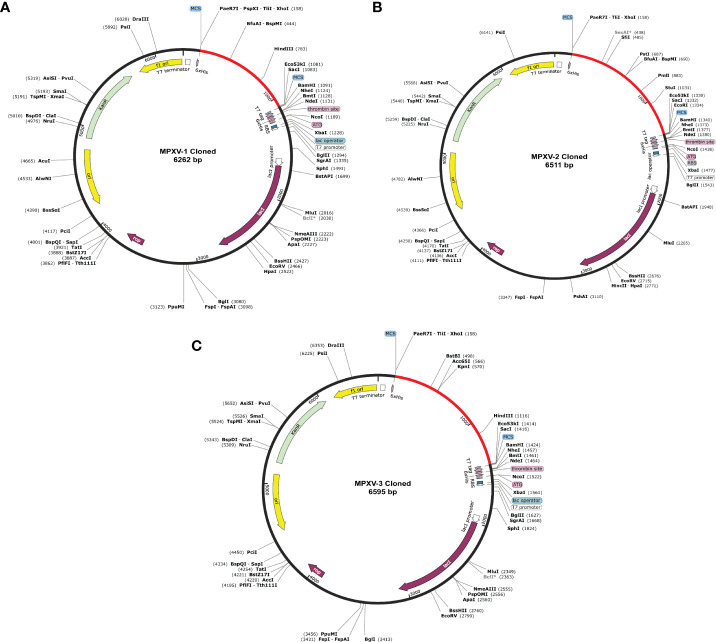
*In silico* cloning of the designed vaccine constructs **(A)** MPXV-1 **(B)** MPXV-2 **(C)** MPXV-3. The optimized DNA sequence of the proposed vaccine construct(s) (shown in red) was cloned between the XhoI and SacI enzyme loci in the expression vector pET-28a (+) (shown in black).

Before cloning, cutting sites of XhoI (CTCGAG at the N-terminal) and SacI (GAGCTC at the C-terminal) were added flanking the constructs’ DNA sequence. The new DNA sequence of MPXV-1–3 was 927, 1176, and 1260 nucleotides **(** **Table S13** and **Figure S7** **).** Using the SnapGene software, the nucleotide sequence of each construct was then inserted between XhoI (site 158) and SacI (site 190) site in the pET-28a (+) vector. The cloned plasmid with MPXV-1–3 construct has a total length of 6262bp, 6511bp, and 6595bp, respectively, as shown in the **Figure 8**. The mRNA secondary structure prediction using the RNAfold program in the case of MPXV-1 yielded optimal secondary structure and centroid secondary structure with a minimum free energy of –298.90 kcal/mol and –213.80 kcal/mol, respectively. In the case of MPXV-2, the minimum free energy of obtained optimal secondary structure was –550.40 kcal/mol, and centroid secondary structure was –443.30 kcal/mol. Besides, the optimal secondary structure and centroid secondary structure of MPXV-3 had minimum free energy of –415.20 kcal/mol and –300.20 kcal/mol, respectively. The graphical output of predicted mRNA secondary structures of MPXV-1–3 is presented in **Figure S8**.

### Computational immune assay

**Figure 9:**
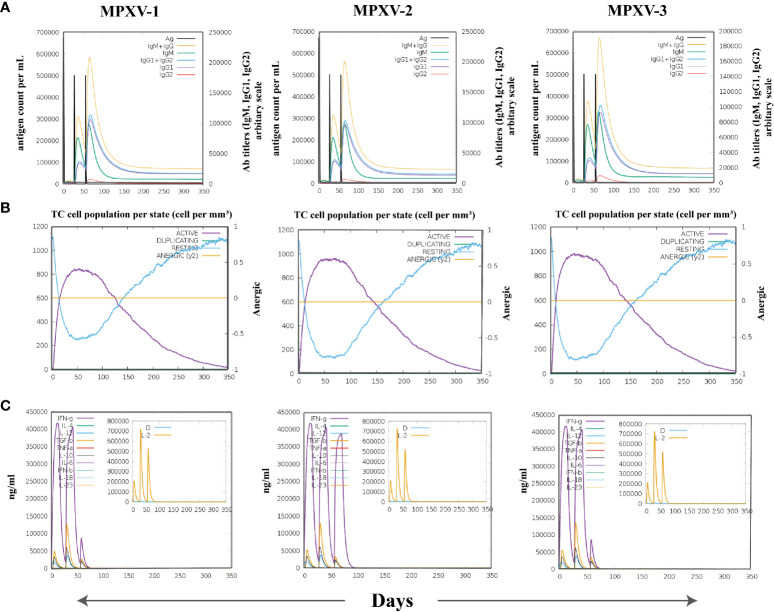
The spectrum of immune stimulated response. **(A)** Antibodies response against antigenic vaccine. Different colours reflected the formation of antibodies that signified the development of the immune response following vaccination and the diverse subtypes of immunoglobulins. **(B)** Cytotoxic T cell population in different states. **(C)** Cell population of cytokine and interleukin in the active and resting states with smaller graph depicting the Simpson index.

The immune simulation conducted by C-ImmSim server showed similar immune response data for the MPXV-1–3 constructs (**Figures 9**, **S9**). The antigen count declined as the antibody level in the immunological response increased, which was primarily attributed to the generation of total B-cells and T cells population. After every injection, there was an increase in the antigen level (>600,000 on 1^st^ and ~500,000 on 2^nd^ and 3^rd^ doses), that subsequently neutralized at the fifth day of injection. This was followed by a rise in the production of secondary immunological responses (IgM + IgG: large titer scale of >200000 per ml) after exposing the host immune system to every vaccine injection. The highest concentration of >200,000 antibody titer per ml was achieved between 54–64 days. As measured by IgM, the primary immune response was similarly elevated to a high degree. Secondary immune responses were followed by tertiary immunological responses, resulting in a large B-cell population, and increasing levels of IgM + IgG, IgM, IgG1 + IgG2, IgG1, and IgG2. The IgM and IgG1 isotype levels rose to 540–560 cells per mm^3^ and 200–220 cells per mm^3^, respectively, and remained there for a long time, concurrently evoking memory cells. Similarly, sustained growth of active CTLs and pre-activation of CTLs response were seen after each immunization. This indicated the long-established immune reaction triggered by designed vaccines. With a peak population of 11000–12000 cells per mm^3^, a substantial number of HTLs stayed active during the simulation, reflecting the generation of an adaptive immune response against the antigen. Additionally, the production of different cytokines, particularly IFN-γ (maximum concentration >400000 ng/ml), was noticed. These findings indicate that the proposed multi-epitope vaccines have the potential to elicit a robust immune response against MPX infection. Nonetheless, *in-vitro*, and *in-vivo* evaluation of the proposed vaccine constructs is recommended to clarify their ability to elicit adaptive immunity against MPXV infection.

### Binding free energy estimation

In thermodynamics calculation, the Gibbs free energy or Delta G (ΔG) (binding affinity of the complex) is vital to estimate the occurrence of a reaction under a cellular environment. To estimate the binding affinity of the MPXV-TLRs complex, MM/GBSA calculations were performed (**Table 7**). The total binding energy (ΔG TOTAL) of MPXV-1/2–TLR3 complex and MPXV-3–TLR2 complex was –103.77, –42.22, and –49.06 kcal/mol, respectively. The high negative ΔG value indicates each complex’s stability and energetically favourable binding. Binding free energy terms, including gas-phase energy (ΔG_gas_ –793.61 kcal/mol) and electrostatic energy (ΔE_elec_ –521.40 kcal/mol), and Van der walls energy (ΔE_vdW_ –272.21 kcal/mol) contributed highest toward total binding energy of MPXV-1–TLR3 complex. In contrast, dominant energy terms in the MPXV-1/2–TLR3 complexes were solvation energy (ΔG _solvation)_ and ΔE_vdW_. ΔE_elec_ of MPXV-1 was negative compared to other constructs due to interactions of charge-charge residues between the vaccine and receptor. This can be attributed to structural adjustments within MPXV-1 exposing the charged residues that possibly established ionic interactions with TLR3. Furthermore, the MPXV-2/3 complex system had high solvation energy than the MPXV-1 system, indicating the structural alterations that likely made the buried regions accessible to solvent.

**Table 7:** MM/GBSA free energy calculations and individual free energy components of vaccine-TLR complex.

Energy Component	MM/GBSA (kcal/mol)					
	MPXV-1–TLR3 complex		MPXV-2–TLR3 complex		MPXV-3–TLR2 complex	
	Average	Std. Err. Mean	Average	Std. Err. Mean	Average	Std. Err. Mean
ΔE_vdW_	–272.21	0.33	–129.54	0.41	–120.97	0.33
ΔE_elec_	–521.40	2.28	322.23	2.77	346.34	2.00
ΔE_GB _	725.66	2.22	–217.95	2.93	–259.64	1.97
ΔE_SURF _	–35.82	0.04	–16.95	0.04	–14.79	0.04
ΔG_gas_	–793.61	2.25	192.68	2.97	225.37	2.02
ΔG_solvation_	689.84	2.21	–234.90	2.90	–274.43	1.96
ΔG_TOTAL_	–103.77	0.29	–42.22	0.25	–49.063	0.20

## Discussion

The choice of appropriate antigens and their immunodominant epitopes is one of the current challenges in the field of multi-epitope vaccine design (17). ViPR comprises B-cell and T-cell epitopes that have undergone experimental validation and were produced from the IEDB for infectious illnesses, allergens, autoimmune disorders, transplant/alloantigens examined in mice, non-human primates, humans, and other animal species (66, 67). This experimental data repository can be exploited to investigate the known epitopes, their immunogenic responses and to formulate a new vaccine (66). In this study, a multi-epitope vaccine was developed for MPXV using the immune epitopes available at ViPR for this virus. Experimentally validated epitopes of MPXV were filtered down to candidate epitopes for vaccine designing by applying several *in silico* tools. Previously, Ismail et al. (68) evaluated the experimentally validated epitopes of hantavirus to shortlist the candidates epitopes for multi-epitope-based vaccine conception.

The antigens of origin of half of the selected epitopes appeared annotated as viral late transcription factor 1, which is an integral membrane component that regulates the DNA-templated transcription. Another antigen is essential IMV membrane protein (A15L), a major component of mature virion membrane necessary for membrane biogenesis. MIR [Monkeypox virus (strain Zaire-96-I-16)] and B6R (negative regulation of complement activation) are also integral membrane components of the virion. A single epitope antigen is a putative enzyme, DNA-directed RNA polymerase 7kDa subunit, with an intracellular localization that assists the synthesis of an RNA transcript from a DNA template. The host’s immune system is more likely to interact with molecules found on the surface of microorganisms, such as plasma membrane proteins, which also play crucial roles in pathogen homeostasis (69). The membrane proteins of the vaccinia virus have been assessed as a part of subunit vaccines and target of neutralizing antibodies *in-vivo* and *in-vitro* (70).

The sequence’s antigenicity prediction is essential because it reveals whether the epitope sequence is likely to be recognized by immunogenic cells in the human body. Another significant barrier to vaccine development is the possibility of allergenicity, which occurs when numerous vaccines drive the immune system into an allergic response (71). Thus, herein, we chose epitopes predicted to be highly antigenic and did not show allergenic potential for the host immune system. On practical grounds, the findings that virulence factors are often the targets of effective immune response could drive the designing of vaccines. Although this notion works well for bacterial pathogens, it can also be suitable for viruses (72). Therefore, the virulence potential of epitopes was tested, and those predicted as virulent were retained. Besides, the non-toxicity of selected epitopes indicates their unharmful effects on the human body (73).

A critical factor in prioritizing the epitope was avoiding any potential cross-reactivity with the host. In general, the least the sequence identity, the higher the likelihood of preventing any possible cross-reactivity. Nonetheless, no research has been done to determine the sequence similarity percentage that may prevent cross-reactivity between the recognition of self-peptides and epitopes (74). When it comes to lengthy epitopes like CD4+ T cell and B-cell epitopes, a 70% cut-off has been defined as conservative by a strategy intended to reduce epitope redundancy (75). In terms of nonameric CD8+ T cell epitopes, it has been noted that residue positions 2 and 9 are crucial for determining epitope binding to MHC-I. In contrast, residues 3-6 and 8 are involved in TCR motif recognition (76). Following Michel-Todó et al. (77), a threshold of 70% could limit the selection of any MPXV epitopes similar to human peptides in more than six (out of nine) residues. Thus, at least one of those crucial places will always be different in any of the CD8+ T cell epitopes from any peptide sequence of that length found in humans. However, as previously stated, cross-recognition may still occur with little similarity; therefore, any possible cross-reactivity must be managed appropriately (77). As structural understanding of the epitope recognition mechanism improves, we think computational methods will soon be able to predict this more accurately. Besides non-homology, the prioritized epitopes sequences showed 100% conservancy with their source proteins sequences from reference MPXV Zaire 77-0666 (NCBI Reference Sequence: NC_003310.1) and MPXV (NCBI Reference Sequence: NC_063383.1). The idea is that a single vaccine would cover the virus’ vast phylogenetic and geographic range. In addition, favourable physicochemical properties of selected epitopes reveal their suitability for application in wet-lab investigations. When choosing the antigens or epitopes that will be included in the vaccine, computational tools can help predict vaccine coverage. Since experimental HLA-restriction alleles of each CTL epitope are reported, we first evaluated whether the IEDB SMM method predicts the reported alleles as high-affinity binders of the respective epitopes. The employed method not only correctly estimated the reported HLA alleles as high-affinity CTL epitopes binders but also revealed additional potent epitopes binders (HLA alleles). Hence, we considered all reported and predicted HLA alleles of CTL epitopes for the population coverage analysis *via* the IEDB tool. The prioritized epitopes were predicted to cover a broad range of the world population (>80% coverage).

Recently, Rawal et al. (25) introduced a new computational system (Vax-ELAN pipeline) to evaluate novel vaccine targets that could act as potential candidates for multi-epitope vaccine construction. They screened the genomic and proteomic datasets of *Vibrio cholerae*, *Plasmodium falciparum*, and *Trypanosoma cruzi* to extract the PVC. Amongst these pathogens, *T. cruzi* proteome was subjected to thorough analysis to shortlist eight proteins as PVC using several properties, for example surface exposed/secretory proteins, virulence, least number of TM helices, antigenicity, non-allergenicity, gene essentiality, non-homology with human proteins and gut microbiota. Further, the researcher subjected the shortlisted proteins to B-cell and T-cell epitopes mapping in order to eventually design a putative multiple epitope vaccine candidate for *T. cruzi.* In the current study, we used Vax-ELAN pipeline together with other immunoinformatics tools to evaluate and shortlist eight MPXV proteins considering the exposed nature, non-allergen, antigen, least number of TM helices (≤1), non-homology, and suitable physiochemical profile. The finalized MPXV proteins (PVC) can be used to map novel B-cell and T-cell epitopes and design a multi-peptide-based vaccine against this virus.

Subunit vaccines need to be administered with an adjuvant because, although safer, they are often less immunogenic and efficacious. Adjuvants are thus crucial for augmenting and guiding the adaptive immune response to vaccine antigens (78). Based on literature mining (68, 79, 80), we used three types of adjuvants to construct MPXV vaccines, including beta-defensin 3, ribosomal protein, and HBHA. Human beta-defensin 3 is an antimicrobial peptide that can play an important role as an immunomodulator to activate human antigen-presenting cells (APCs) (81). 50S ribosomal protein L7/L12 has been shown to induce the maturation of dendritic cells (DCs), CD4+, CD8+, and IFN-producing cells following the stimulation of naïve T cells (21). HBHA is a novel TLR4 agonist with no systemic toxicity. It has a strong immunostimulatory potential and the ability to stimulate the maturation of DCs in a TLR4-dependent manner (82). Following Robert et al. (18), CTL and B-cell epitopes were linked together by inserting AAY and GPGPG linker. An EAAAK and GGGS linker (provides flexible distribution) were added to link the PADRE sequence and an adjuvant and separate the epitope class, respectively, as previously reported (32). The TAT sequence can transport the macromolecular substances and makes the cell membrane penetration easier to stimulate the phagocytosis of vaccines by APCs. Therefore, the TAT sequence was incorporated at the N-termini of constructed vaccines (83).

The proposed vaccines in this study had reasonable molecular weight, i.e., less than 110kDa, which hinted that it is suitable for application (84). The estimated half-life in *E. coli* (10 hrs), instability index (19.39 – 27.43), and aliphatic index (84.02 – 92.99) of the vaccine constructs point towards the usage of this organism for heterologous expression. That is why we optimized the codon of constructs based on *E. coli* (strain K12) and carried out computational cloning in pET28a (+), a common expression vector. Furthermore, the predicted pI indicated that MPXV-1 (pI=7.66) would assume a positive charge under neutral pH conditions, while MPXV-2/3 (pI= 4.68 and 4.70, respectively) would be negatively charged. The negative net charge leans towards assisting in (Ni^2+^) affinity purification procedure, like those carried in Nickel immobilized columns (85, 86). Further, considerable structural alterations in protein are unlikely to happen in such pH conditions. The successful manufacturing process is verified by solubility predictions of all designed vaccines. The secondary structure analysis showed that the vaccine proteins have 33% to 48% random coil. Since the spatial structure of random coil is loose and it is easy to form epitopes; thus, the modeled vaccines have fine structural basis because they contain large amount of this structure. Hashemzadeh et al. (87), Rekik et al. (88), and Droppa-Almeida et al. (89) predicted tertiary structure of constructed vaccine with a Z-score of −2.11 kcal/mol, −9.5 kcal/mol, and −5.26 kcal/mol, respectively. They concluded that the predicted structures were reliable and of good quality. In the present study, the predicted tertiary structures of proposed vaccines (Z-score of MPXV-1–3 = −4.25, −5.62, and −5.87) resemble the structural properties of similar-sized proteins determined by the X-Ray crystallography.

When microorganisms break through the mucosal barrier, TLR can recognize them and initiate the adaptive immune response (84). Because the TLR3 molecule plays a vital role in innate immunity against the virus, the binding of antigen and TLR3 can help APCs in antigen presentation and release of local cytokines. Data also suggest that TLR3 can detect certain DNA viruses (90). We conducted protein-protein docking to prove that the designed vaccines had a strong affinity with the TLR3 receptor (79). The results showed the lowest energy value (more stable) for vaccine-TLR3 complexes. Further, ionic and hydrogen bonding also indirectly supported the stable binding between vaccine constructs and TLR3 (91). MPXV-1/2 had high docking energy with TLR3 and therefore was subjected to additional analysis. We also performed docking of designed vaccines with TLR2 and TLR4 receptors, which were previously studied in the context of their role in viral recognition and induction of inflammatory cytokines (92). All vaccine proteins demonstrated reasonable docking energy with these immune receptors (with several intermolecular interactions), implying their potential to stimulate the downstream immune responses. Interestingly, MPXV-3 indicated higher docking energy with TLR2 than other receptors; hence was recommended for further investigations.

Coherently, RMSD measures obtained by molecular dynamic simulation have indicated stable interaction between the ligand and the receptor. The agility and compactness of the vaccine-TLRs complex structure were also supported by the RMSF and Rg profile of the complexes. Moreover, structural adjustments in vaccine protein structure upon binding TLRs were revealed by FEL and SASA descriptors, which seem crucial to gathering stability for exposure to the immune system. H-bond analysis showed persistent H-bonds between vaccine and TLRs across the simulation, which is critical for complex stability (93). Consistent with the docking analysis, MM/GBSA estimations validated a high binding affinity (low negative ΔG values) of vaccine-TLR2/3 complexes.

A relevant antibodies generation after vaccination was predicted by the C-ImmSim server, which is also regarded as crucial for preventing infection. Memory B-cells can present antigens on the cell surface, activate HTLs, and serve as the subsequent target of effector HTLs in secondary antibody responses (94). Immune simulation prediction in this study also revealed significant memory B-cells production, which has appeared to engage HTLs as evident by their high numbers. The generation of HTLs is also attributed to GPGPG linkers added to vaccine constructs. Besides, the secretion of IFN-γ, long-lasting cellular and CTL response was detected during the immune response. *In silico* immune simulation results of the present study are comparable to previous investigations that used B-cell, CTL, and HTL epitopes in vaccine construction (21, 32, 95). The data highlights the potential of designed vaccine constructs to induce a robust immune response and provide protection against MPXV infection. To confirm the results of the current study, however, experimental validation of the constructed vaccines is warranted.

## Limitations

This study highlighted an alternative vaccine design approach based on a multi-epitope ensemble of the antigenic proteins of the MPX genome to handle the antigenic complexity. The constructed vaccines were recommended based on immunoinformatics-guided evaluation and are believed to be immunogenic; however, the extent of protection from MPX infection is unknown. Concerning T cell components, a population coverage estimation was based on a binding prediction of peptide-MHC molecules. Although the reliability of peptide-MHC affinity predictions has been extensively proved, this feature will have to be tested further. A suitable antigen processing is a vital feature for the immunogenicity of epitopes, and this would need thorough evaluation in the context of epitope delivery in the form of a genetic construct. The ordering and spacing of the CD8+ T cell and B-cell epitopes herein were provided following the literature. Still, this would require more proof to determine the optimal ordering and spacing to acquire the best immunogenicity with CTL and B-cell epitopes. Omics database repository, including ViPR, and several immunoinformatics tools described in the manuscript, are extremely useful for carrying out *in silico* studies that can guide the web-lab experiments, contributing to time and money-saving. However, *in-vitro* immunological studies will be the next step to ascertain the designed vaccines’ immunogenicity and design challenge-protection preclinical experiments to ultimately validate the strategy.

## Conclusion

Multi-epitope vaccines have gained significant attention, with many showing protective efficacy *in-vitro* and progressed to clinical trials. The goal of the present study was to propose an alternative approach to prioritize the experimentally determined epitopes of MPXV for inclusion in designing a multi-epitope-based vaccine through an immunoinformatics-driven approach. The physicochemical and antigenic properties of constructed vaccines were analyzed computationally. The stability profile and molecular interactions between the proposed vaccines and immunological receptors were examined using molecular docking and molecular dynamics simulations. Binding free energy calculations confirmed the stable and energetically favourable binding of MPXV-1−TLR3 (-103.7705 kcal/mol), MPXV-2−TLR3 (-42.2205 kcal/mol), and MPXV-3−TLR2 (-49.0633 kcal/mol). The proposed ensembles’ potential to evoke cellular and humoral immunity against the virus was demonstrated by computational immune simulation. Using various immunoinformatics and *in silico*-based methods, protective vaccines against viral infection were developed and evaluated herein; nevertheless, experimental testing is required to ascertain the vaccine efficacy. Additionally, we encourage further research on the production and biological activities of the developed multi-epitope vaccines.

## Data availability statement

The original contributions presented in the study are included in the article/**Supplementary Material**. Further inquiries can be directed to the corresponding authors.

## Author contributions

SA, FA and AA conceived and designed the study. SA, MW and FA performed experiments. MA, IU and NK analyzed the data. SA, MW, MA and AA wrote the manuscript with inputs and comments from all co-authors. All authors contributed to the article and approved the submitted version.

## Funding

The authors thank the Researchers Supporting Project number (RSP-2021/198), King Saud University, Riyadh, Saudi Arabia.

## Conflict of interest

The authors declare that the research was conducted in the absence of any commercial or financial relationships that could be construed as a potential conflict of interest.

## Publisher’s note

All claims expressed in this article are solely those of the authors and do not necessarily represent those of their affiliated organizations, or those of the publisher, the editors and the reviewers. Any product that may be evaluated in this article, or claim that may be made by its manufacturer, is not guaranteed or endorsed by the publisher.

## Abbreviations

CTL, Cytotoxic T lymphocytes; FEL, free energy landscape; MHC, major histocompatibility complex; MM-GBSA, Molecular mechanics/generalized born surface area; MPX, monkeypox; MPXV, Monkeypox virus; Rg, radius of gyration; RMSD, root mean square deviation; RMSF, root mean square fluctuation; SASA, solvent accessible surface area; TLR, toll-like immune receptors.
