# Supplementary material for: Contriving multi-epitope vaccine ensemble for monkeypox disease using an immunoinformatics approach
Source: Front Immunol. 2022 Oct 13;13:1004804. doi: 10.3389/fimmu.2022.1004804 (PMC9606759; doi:10.3389/fimmu.2022.1004804)
Supplement: Supplementary file 1 [file DataSheet_1.docx]

**Contriving Multi-Epitope Vaccine Ensemble for Monkeypox Disease using an Immunoinformtics Approach**

**Shahkaar Aziz^1†,^ Fahad Nasser Almajhdi^2†,^ Muhammad Waqas^3,4*^, Inam Ullah^3^, Muhammad Adil Salim^5,6^, Nasir Ali Khan^4^, Amjad Ali^3*^**

^1^Institute of Biotechnology and Genetic Engineering, University of Agriculture Peshawar, Peshawar 25000, Pakistan.

^2^Department of Botany and Microbiology, College of Science, King Saud University, Riyadh 11451, Saudi Arabia.

^3^Department of Biotechnology and genetic Engineering, Hazara University, Mansehra 21120, Pakistan.

^4^Natural and Medical Sciences Research Center, University of Nizwa, Birkat-ul-Mouz 616, Nizwa, Oman.

^5^Microbiology Graduate Group, University of California, Davis, Davis, CA, United States.

^6^Genome Center, University of California, Davis, Davis, CA, United States,

*** Correspondence:**

Muhammad Waqas

mwaqas@unizwa.edu.om

Amjad Ali

amjad.genetics@hu.edu.pk

^†^ = These authors equally contributed

**Table of contents**

| **Table S1.** | Antigen source and entry details of finalized epitopes for MPXV vaccine design |
| --- | --- |
| **Table S2** | Best-ranked out membrane proteins of MPXV obtained following Vax-ELAN pipeline |
| **Table S3.** | MHC-I restricted alleles prediction for selected MHC I epitopes by IEDB SMM method. Letter in bold indicates experimentally validated restricted allele for the respective epitope also predicted by IEDB server. |
| **Table S4*.*** | GalaxyRefine output for the submitted initial model of vaccine constructs predicted by the RoseTTAFold server. (Letter in bold indicates the final model selected for further analysis). |
| **Table S5.** | Molecular docking calculations and interactions details between selected CTL epitopes and HLA-A*0201 allele (MOE2020). H, hydrogen bond; IH, Ionic interaction; A, Arene interaction |
| **Table S6.** | Molecular docking calculations and interactions details between selected CTL epitopes and HLA-B*15:02 allele (MOE2020). H, hydrogen bond; IH, Ionic interaction; A, Arene interaction |
| **Table S7.** | Atom-atom interactions across TLR3-MPXV-1 interface obtained using PDBsum server |
| **Table S8.** | Atom-atom interactions across TLR3-MPXV-2 interface obtained using PDBsum server |
| **Table S9.** | Atom-atom interactions across TLR2-MPXV-3 interface obtained using PDBsum server |
| **Table S10.** | Hydrogen bonds at the TLR3– MPXV-1 construct interface (with occupancy ≥ 2%) |
| **Table S11.** | Hydrogen bonds at the TLR3– MPXV-2 construct interface (with occupancy ≥ 2%) |
| **Table S12.** | Hydrogen bonds at the TLR2– MPXV-3 construct interface (with occupancy ≥ 2%) |
| **Table S13.** | Complementary DNA sequences of the constructed monkeypox vaccines were subjected to in-silico cloning and RNA structure prediction in this study. The N-terminal contains inserted XhoI restriction site, and the C-terminal contains inserted SacI restriction site (shown in bold letters). |
| **Figure S1.** | Hierarchical representation of Vax-ELAN pipeline followed in selecting best-ranked outer membrane proteins of MPXV. Out of 191 proteins of MPXV, the integrated pipeline finalized eight proteins (A21L, A30L, A43R, B8R, B9R, B20R, C22L, and J2R) as potential vaccine candidates. These proteins can be subjected to novel B-cell and T-cell epitopes mapping. |
| **Figure S2.** | Conformational B-cell epitopes predicted in MPXV-1. The light-yellow spheres showing epitopes containing **(A)** 15 residues (AA 273-287) with score 0.769 **(B)** 43 residues (AA 89, 92-134) with score 0.759 **(C)** 32 residues (AA 1-22, 30-39) with score 0.715 **(D)** 31 residues (191-220, 225,226) with score 0.667 **(E)** 36 residues (AA 227-265) with score 0.626 **(F)** 9 residues (AA 167-172, 174, 175, 178) with score 0.621 **(G)** 5 residues (AA267-272) with score 0.552. |
| **Figure S3.** | Conformational B-cell epitopes predicted in MPXV-2. The light-yellow spheres showing epitopes containing **(A)** 45 residues (AA 174-218) with score 0.865 **(B)** 67 residues (AA 52-102, 114-131) with score 0.704 **(C)** 34 residues (AA1-35) with score of 0.671 **(D)** 61 residues (AA277-302, 307-319, 335-344, 347-348, 350-353, 371-375, 377) with score 0.628. |
| **Figure S4.** | Conformational B-cell epitopes predicted in MPXV-3. The light-yellow spheres showing epitopes containing **(A)** 35 residues (AA1-35) with score 0.902 **(B)** 5 residues (AA 222-226) with score 0.835 **(C)** 34 residues (AA384-417) with score 0.828 **(D)** 52 residues (AA82-134) with score 0.772 **(E)** 29 residues (AA207-221, 227-239, 242) with score 0.675 **(F)** 11 residues (AA36-46) with score **(G)** 46 residues (AA267, 270-283, 337, 352-383) with score 0.526 **(H)** 6 residues (AA174-179) with score 0.522. |
| **Figure S5.** | Predominant binding mode of selected CTL epitopes with HLA-A*0201 allele (MOE2020). E1, AIIDIEPDL; E2, IHLEWLLGF; E3, LQKFSFKIA; E4, RTVIHLEWL; E5, SLKDVLVSV; E6, WKVLSIMAF; E7, ITVGMLIYSM; E8, KLSSYHVVSV; E9, NVDSTDELM; E10, WAIIPLSASV. |
| **Figure S6.** | Predominant binding mode of selected CTL epitopes with HLA-B*15:02 allele (MOE2020). E1, AIIDIEPDL; E2, IHLEWLLGF; E3, LQKFSFKIA; E4, RTVIHLEWL; E5, SLKDVLVSV; E6, WKVLSIMAF; E7, ITVGMLIYSM; E8, KLSSYHVVSV; E9, NVDSTDELM; E10, WAIIPLSASV. |
| **Figure S7.** | Optimized cDNA construct of **(A)** MPXV-1 **(B)** MPXV-2 **(C)** MPXV-3 with restriction enzymes sites added used for in silico cloning in the expression vector. |
| **Figure S8.** | Predicted minimum free energy (MFE) mRNA secondary structure **(A)** and centroid mRNA secondary structure **(B)** of MPXV-1–3 (RNAfold program). The MFE structures are colored by base-pairing probabilities. For unpaired regions the color denotes the probability of being unpaired. |
| **Figure S9.** | In silico immune simulation of an infection challenge, comprised of a virus responding to the sequence of MPXV proteins covered by the multi-epitope construct. (A) The total number of B-cell and isotypes per entity state. (B) The cell count of T-helper lymphocytes is shown in various forms, i.e., active, duplicating (in the mitotic cycle), resting (not active), and anergic. |

**Table S1.** Antigen source (Protein name) and entry details of finalized epitopes used for MPXV vaccine construction

| Epitope IEDB ID | Epitopes | Protein Name | UniProt Accession No. of Protein |
| --- | --- | --- | --- |
| 80278 | AIIDIEPDL | EVN088, Late transcription factor VLTF-1, MPXV-WRAIR072, RPXV075, late gene transcription VLTF-1, late gene transcription factor VLTF-1, late gene transcription protein, late transcription factor VLTF-1, viral late transcription factor 1 | Q5IXU7 |
| 83491 | IHLEWLLGF | EVN088, Late transcription factor VLTF-1, MPXV-WRAIR072, RPXV075, late gene transcription VLTF-1, late gene transcription factor VLTF-1, late gene transcription protein, late transcription factor VLTF-1, viral late transcription factor 1 | Q5IXU7 |
| 85726 | LQKFSFKIA | EVN088, Late transcription factor VLTF-1, MPXV-WRAIR072, RPXV075, late gene transcription VLTF-1, late gene transcription factor VLTF-1, late gene transcription protein, late transcription factor VLTF-1, viral late transcription factor 1 | Q5IXU7 |
| 88280 | RTVIHLEWL | EVN088, Late transcription factor VLTF-1, MPXV-WRAIR072, RPXV075, late gene transcription VLTF-1, late gene transcription factor VLTF-1, late gene transcription protein, late transcription factor VLTF-1, viral late transcription factor 1 | Q5IXU7 |
| 112322 | SLKDVLVSV | CMP80R, CPXV093 protein, DNA-dependent RNA polymerase subunit rpo7, EVN085, HSPV084, MPXV-WRAIR069, RNA polymerase, RNA polymerase 7 subunit, RNA polymerase subunit RPO7, RPXV072, putative EVM067 protein | M1LBN6 |
| 90583S | WKVLSIMAF | A15L, CPXV146 protein, HSPV134, MPXV-WRAIR119, RPXV122, phosphorylated IMV membrane protein | Q3I841 |
| 29131 | ITVGMLIYSM | A15L, CPXV146 protein, HSPV134, MPXV-WRAIR119, RPXV122, phosphorylated IMV membrane protein | Q3I841 |
| 84525 | KLSSYHVVSV | EVN088, Late transcription factor VLTF-1, MPXV-WRAIR072, RPXV075, late gene transcription VLTF-1, late gene transcription factor VLTF-1, late gene transcription protein, late transcription factor VLTF-1, viral late transcription factor 1 | Q5IXU7 |
| 87120 | NVDSTDELM | EVN088, Late transcription factor VLTF-1, MPXV-WRAIR072, RPXV075, late gene transcription VLTF-1, late gene transcription factor VLTF-1, late gene transcription protein, late transcription factor VLTF-1, viral late transcription factor 1 | Q5IXU7 |
| 90544 | WAIIPLSASV | EVN088, Late transcription factor VLTF-1, MPXV-WRAIR072, RPXV075, late gene transcription VLTF-1, late gene transcription factor VLTF-1, late gene transcription protein, late transcription factor VLTF-1, viral late transcription factor 1 | Q5IXU7 |
| 39258 | *LSAATETYSGLTPEQKAYVPAMF | M1R IMV membrane protein, IMV membrane protein target of neutralizing antibody, M1R, MPXV-COP-074, MPXV-SL-074, MPXV-WRAIR074, MPXVgp080, Myristylprotein | Q8V502 |
| 10135 | *DSGYHSLDPNAVCETD | B6R, EEV membrane glycoprotein, EEV type-I membrane glycoprotein, MPXV-COP-159, MPXV-WRAIR159 | Q773E2 |
| 73976 | *YGAPGSPTNLEFINTGSSK | M1R | Q8V502 |
| 7309 | *CVRSNEEFDPVDDGPDDETDLSKLSKD | B6R, EEV membrane glycoprotein, EEV type-I membrane glycoprotein, MPXV-COP-159, MPXV-WRAIR159 | Q773E2 |
|  | *B-cell epitopes | | |

**Table S2.** Best-ranked out membrane proteins of MPXV obtained following Vax-ELAN pipeline

| **NCBI ID** | **Locus** | **Protein Name** | **Location** | **Non-homology** | **Non allergen** | **adhesion** | **MW** | **Stability** | **VexiJen** | **TM Helics** |
| --- | --- | --- | --- | --- | --- | --- | --- | --- | --- | --- |
| NP_536475.1 | C22L | Protein F16 | Extra | Yes | Yes | adhesive | 15725.32 | 35.11 | 0.4123 | 1 |
| NP_536558.1 | A21L | Virion membrane protein A21 | Extra | Yes | Yes | adhesive | 13385.45 | 31.99 | 0.4354 | 0 |
| NP_536567.1 | A30L | IMV phosphoprotein, IMV protein, IMV protein for association of denseviroplasm with viral membrane during morphogenesis, IMV protein for association of denseviroplasm with viral membranes during morphogenesis, MV protein, Protein A30, VACV-DUKE-161, VACV149, Virion morphogenesis protein, Virion protein A30L | Extra | Yes | Yes | adhesive | 16403.49 | 29.97 | 0.5673 | 0 |
| NP_536596.1 | B8R | Bifunctional 21 kDa protein of 18 kDa membrane protein, Bifunctional 21kDa protein of 18kDa membrane protein/deletion reduces lesion in VAC-mouse skin model, MPXV-COP-161, MPXV-SL-161, MPXV-WRAIR161, MPXVgp169, Virulence, ER resident | Extra | Yes | Yes | adhesive | 21487.924 | 29.33 | 0.4362 | 0 |
| NP_536597.1 | B9R | B9R, MPXV-COP-162, MPXV-SL-162, MPXV-WRAIR162, MPXVgp170, Soluble interferon-gamma receptor-like protein | Extra | Yes | Yes | adhesive | 30533.29 | 37.16 | 0.4803 | 0 |
| NP_536608.1 | B20R | Uncharacterized protein | Extra | Yes | Yes | adhesive | 21566.3972 | 33.69 | 0.574 | 0 |
| NP_536617.1 | J2R | MPXV-COP-002, MPXV-COP-176, MPXV-WRAIR002, MPXV-WRAIR176, MPXVgp002, MPXVgp190, TNF receptor (CrmB), TNF-alpha-receptor-like protein, Tumor necrosis factor receptor II homolog | Extra | Yes | Yes | adhesive | 38212.2892 | 34.91 | 0.5154 | 0 |
| NP_536580.1 | A43R | A43R, Putative type-I membrane glycoprotein, Type I membrane glycoprotein | Extra | Yes | Yes | adhesive | 23085.7033 | 30.46 | 0.5089 | 1 |

**Table S3.** MHC-I restricted alleles prediction for selected MHC I epitopes by the IEDB SMM method. The letter in bold indicates the experimentally validated restricted allele for the respective epitope also predicted by the IEDB server.

| **Epitope (Host)** | **IEDB Predicted MHC I Restricted Allele** | **VIPR Database** |
| --- | --- | --- |
| AIIDIEPDL (Human) | **HLA-A*02:01** (426.69), HLA-A*02:06 (530.33), HLA-B*15:02 (529.94) | HLA-A*02:01 |
| IHLEWLLGF (Monkey) | HLA-B*15:02 (251.90), HLA-A*24:02 (453.56) | Mamu-B*017:04 |
| LQKFSFKIA (Human) | HLA-A*30:01 (161.80), **HLA-A*02:01** (156.87), HLA-A*02:06 (117.39) HLA-B*15:02(114.12), | HLA-A*02:01 |
| SLKDVLVSV (Human) | **HLA-A*02:01** (44.17), HLA-A*02:06 (47.81), **HLA-C*07:02** (161.99), HLA-A*30:01 (59.88) | HLA-A*02:01,  HLA-C*07:02 |
| RTVIHLEWL (Human) | HLA-A*02:06 (201.16), HLA-A*30:01 (129.09), **HLA-A*02:01** (252.12), HLA-B*15:02(147.99), HLA-C*07:02 (77.98) | HLA-A*02:01 |
| ITVGMLIYSM  (Human) | **HLA-A*02:06** (452.43), **HLA-A*02:02** (432.56), **HLA-A*68:02** (601.99), **HLA-A*02:03** (219.05), HLA-B*15:01 (94.89), HLA**-A*02:01** (157.76), HLA-B*15:02 (465.85) | HLA-A*02:01, HLA-A*02:02, HLA-A*02:06, HLA-A*68:02, HLA-A*02:03 |
| NVDSTDELM(Human) | **HLA-A*02:01** (181.76), HLA-A*01:01 (147.04), HLA-C*07:02 (92.72), HLA-B*15:02 (170.70) | HLA-A*02:01 |
| KLSSYHVVSV(Human) | **HLA-A*02:01** (114.0), HLA-A*02:06 (169.65), HLA-A*68:02 (210.18), HLA-C*07:02 (229.18), HLA-B*58:02 (83.53) | HLA-A*02:01 |
| WAIIPLSASV(Human) | HLA-A*02:06 (19.89), **HLA-A*02:01** (94.21), HLA-B*15:02 (168.74), HLA-A*68:02 (185.61), HLA-B*15:01 (64.63) | HLA-A*02:01 |
| WKVLSIMAF(Human) | HLA-C*07:02 (361.55), HLA-B*15:01 (15.55), **HLA-B*15:03** (32.68) | HLA-B*15:03 |

**Table S4.** GalaxyRefine output for the submitted initial model of vaccine constructs predicted by the RoseTTAFold server. (Letter in bold indicates the final model selected for further analysis).

| Vaccine | Model | GDT-HA | RMSD | MolProbity | Clash score | Poor | Model |
| --- | --- | --- | --- | --- | --- | --- | --- |
| MPXV-1 | Initial | 1.0000 | 0.000 | 1.367 | 1.5 | 0.0 | 92.4 |
|  | MODEL 1 | 0.9845 | 0.305 | 1.877 | 10.4 | 0.4 | 95.1 |
|  | **MODEL 2** | 0.9730 | 0.330 | 1.869 | 10.2 | 0.4 | 95.1 |
|  | MODEL 3 | 0.9763 | 0.326 | 1.845 | 9.1 | 0.4 | 94.7 |
|  | MODEL 4 | 0.9845 | 0.307 | 1.890 | 9.7 | 0.0 | 94.4 |
|  | MODEL 5 | 0.9812 | 0.314 | 1.897 | 10.4 | 0.4 | 94.7 |
| MPXV-2 | Initial | 1.0000 | 0.000 | 1.440 | 2.0 | 0.0 | 92.5 |
|  | MODEL 1 | 0.9955 | 0.251 | 1.940 | 9.6 | 0.4 | 93.3 |
|  | MODEL 2 | 0.9916 | 0.256 | 1.975 | 10.5 | 0.4 | 93.3 |
|  | MODEL 3 | 0.9955 | 0.249 | 1.947 | 9.8 | 0.4 | 93.3 |
|  | MODEL 4 | 0.9923 | 0.262 | 2.012 | 11.9 | 0.0 | 93.5 |
|  | **MODEL 5** | 0.9897 | 0.264 | 1.988 | 11.2 | 0.0 | 93.5 |
| MPXV-3 | Initial | 1.0000 | 0.000 | 1.349 | 2.2 | 0.0 | 94.9 |
|  | MODEL 1 | 0.9838 | 0.297 | 1.746 | 9.1 | 0.3 | 96.1 |
|  | MODEL 2 | 0.9892 | 0.281 | 1.693 | 7.5 | 0.3 | 95.9 |
|  | MODEL 3 | 0.9820 | 0.301 | 1.757 | 8.4 | 0.3 | 95.7 |
|  | MODEL 4 | 0.9832 | 0.300 | 1.696 | 8.0 | 0.3 | 96.1 |
|  | **MODEL 5** | 0.9868 | 0.277 | 1.686 | 8.8 | 0.3 | 96.6 |

**Table S5.** Molecular docking calculations and interaction details between selected CTL epitopes and HLA-A*0201 allele (MOE2020). H, hydrogen bond; IH, Ionic interaction; A, Arene interaction

| **Epitope No.** | **Docking score (kcal/mol)**  **Receptor: HLA-A*0201** | **Bond Type** | **4U6X** | **Epitope Residues** | **Energy (Kcal/mol)** | **Distance(Å)** | **Backbone Interaction** | **Bond Frequency** |
| --- | --- | --- | --- | --- | --- | --- | --- | --- |
| AIIDIEPDL | -10.2862 | IH | Arg65 | Glu6 | -37.65 | 2.96 | -- | 6 |
|  |  | H | Lys66 | Asp8 | -7.10 | 2.75 | -b | 1 |
|  |  | H | His70 | Pro7 | -4.80 | 2.73 | -b | 1 |
|  |  | H | Thr73 | Asp4 | -2.30 | 2.85 | -b | 1 |
|  |  | IH | Asp77 | Ala1 | -25.71 | 2.75 | -b | 3 |
|  |  | H | Asp77 | Ile2 | -3.10 | 3.01 | -b | 1 |
|  |  | H | Thr143 | Ala1 | -1.40 | 2.97 | -b | 1 |
|  |  | A | Trp147 | Ala1 | -0.70 | 3.58 | -- | 1 |
|  |  | H | Gln155 | Ile5 | -2.60 | 2.86 | -b | 1 |
|  |  | H | Gln155 | Asp8 | -4.30 | 2.82 | -- | 1 |
|  |  | H | Tyr159 | Leu9 | -2.70 | 2.77 | -- | 1 |
| IHLEWLLGF | -10.6489 | H | Glu63 | Gly8 | -9.00 | 2.96 | -b | 2 |
|  |  | IH | Arg65 | Phe9 | -16.95 | 3.06 | -- | 3 |
|  |  | H | Lys66 | Leu6 | -11.00 | 2.74 | -b | 1 |
|  |  | IH | Lys66 | Phe9 | -19.64 | 2.69 | -- | 2 |
|  |  | H | Thr73 | Trp5 | -2.20 | 2.90 | -- | 1 |
|  |  | A | Tyr99 | Leu6 | -0.60 | 3.63 | -- | 1 |
|  |  | H | Glu154 | Ile1 | -5.50 | 2.73 | bb | 1 |
|  |  | H | Gln155 | Ile1 | -7.10 | 2.79 | bb | 1 |
|  |  | H | Gln155 | Leu3 | -1.60 | 2.90 | -b | 1 |
|  |  | H | Tyr159 | Leu7 | -4.10 | 2.66 | -b | 1 |
|  |  | H | Trp167 | Phe9 | -4.90 | 2.83 | -- | 1 |
| LQKFSFKIA | -10.1632 | IH | Glu63 | Leu1 | -22.70 | 3.09 | -b | 4 |
|  |  | H | Lys66 | Gln2 | -9.70 | 2.75 | -- | 1 |
|  |  | H | Thr73 | Ile8 | -1.40 | 2.75 | -b | 1 |
|  |  | H | Asp77 | Ala9 | -1.40 | 2.44 | -b | 1 |
|  |  | H | Tyr99 | Leu1 | -2.60 | 3.02 | -b | 1 |
|  |  | A | Trp147 | Lys7 | -0.60 | 3.62 | -- | 1 |
|  |  | H | Gln155 | Lys3 | -2.30 | 2.84 | -b | 1 |
|  |  | H | Gln155 | Ser5 | -1.50 | 2.92 | -b | 1 |
|  |  | H | Gln155 | Phe6 | -4.10 | 2.83 | -b | 1 |
|  |  | H | Gln155 | Lys7 | -4.60 | 3.00 | -b | 1 |
| RTVIHLEWL | -11.1373 | H | Arg65 | Leu6 | -9.70 | 2.85 | -b | 2 |
|  |  | IH | Lys66 | Leu9 | -13.77 | 2.69 | -b | 2 |
|  |  | H | His70 | Glu7 | -1.80 | 2.79 | -- | 1 |
|  |  | IH | Asp77 | Arg1 | -43.36 | 2.97 | -* | 9 |
|  |  | H | Thr80 | Arg1 | -5.70 | 2.84 | -b | 1 |
|  |  | H | Arg97 | His5 | -3.90 | 3.09 | -- | 2 |
|  |  | A | Tyr116 | Arg1 | -0.80 | 3.34 | -- | 1 |
|  |  | H | Thr143 | Arg1 | -2.70 | 3.12 | -- | 1 |
|  |  | H | Lys146 | Arg1 | -8.60 | 2.96 | -b | 1 |
|  |  | H | Gln155 | Ile4 | -3.80 | 2.78 | -b | 1 |
|  |  | H | Tyr159 | Trp8 | -1.10 | 2.90 | -b | 1 |
| SLKDVLVSV | -9.73509 | IH | Lys66 | Val9 | -19.56 | 2.81 | -- | 3 |
|  |  | H | Thr73 | Val5 | -2.00 | 2.93 | -b | 1 |
|  |  | H | Lys146 | Leu2 | -8.60 | 2.82 | -b | 1 |
|  |  | H | Trp147 | Leu2 | -2.80 | 2.95 | -b | 1 |
|  |  | A | Trp147 | Lys3 | -0.50 | 4.57 | -b | 1 |
|  |  | H | Gln155 | Lys3 | -9.50 | 2.68 | -- | 1 |
|  |  | H | Gln155 | Asp4 | -2.00 | 3.32 | -b | 1 |
|  |  | H | Tyr159 | Val9 | -2.10 | 2.66 | -- | 1 |
|  |  | H | Trp167 | Val9 | -4.60 | 2.79 | -- | 1 |
| WKVLSIMAF | -9.43655 | IH | Glu63 | Lys2 | -23.07 | 2.78 | -- | 4 |
|  |  | H | Thr80 | Phe9 | -2.50 | 2.66 | -- | 1 |
|  |  | H | Tyr84 | Phe9 | -4.80 | 2.58 | -- | 1 |
|  |  | IH | Lys146 | Phe9 | -22.32 | 2.69 | -- | 2 |
|  |  | H | Trp147 | Ala8 | -2.30 | 2.86 | -b | 1 |
|  |  | A | Trp147 | Phe9 | -0.50 | 4.76 | -- | 1 |
|  |  | H | Gln155 | Trp1 | -5.40 | 2.73 | bb | 1 |
|  |  | H | Gln155 | Ile6 | -1.30 | 3.00 | -b | 1 |
|  |  | A | Tyr159 | Lys2 | -0.70 | 3.63 | -- | 1 |
| ITVGMLIYSM | -10.7761 | H | Lys66 | Thr2 | -1.00 | 2.75 | -b | 1 |
|  |  | H | Lys66 | Val3 | -1.70 | 3.16 | -b | 1 |
|  |  | H | Lys66 | Met5 | -0.50 | 3.95 | b- | 1 |
|  |  | A | His70 | Met5 | -1.00 | 3.85 | -b | 1 |
|  |  | H | Thr73 | Ile7 | -2.70 | 2.75 | -b | 1 |
|  |  | H | Asp77 | Met10 | -0.60 | 4.02 | b- | 1 |
|  |  | H | Tyr84 | Met10 | -2.90 | 2.65 | -b | 1 |
|  |  | H | Arg97 | Met5 | -3.50 | 2.97 | -b | 1 |
|  |  | H | Thr143 | Met10 | -2.60 | 2.90 | -b | 1 |
|  |  | IH | Lys146 | Met10 | -15.44 | 2.85 | -b | 3 |
|  |  | H | Trp147 | Met10 | -3.10 | 2.81 | -b | 1 |
|  |  | H | Gln155 | Leu6 | -3.90 | 2.81 | -b | 1 |
| KLSSYHVVSV | -10.2734 | H | Lys66 | Thr2 | -1.00 | 2.75 | -b | 1 |
|  |  | H | Lys66 | Val3 | -1.70 | 3.16 | -b | 1 |
|  |  | H | Lys66 | Met5 | -0.50 | 3.95 | b- | 1 |
|  |  | A | His70 | Met5 | -1.00 | 3.85 | -b | 1 |
|  |  | H | Thr73 | Ile7 | -2.70 | 2.75 | -b | 1 |
|  |  | H | Asp77 | Met10 | -0.60 | 4.02 | b- | 1 |
|  |  | H | Tyr84 | Met10 | -2.90 | 2.65 | -b | 1 |
|  |  | H | Arg97 | Met5 | -3.50 | 2.97 | -b | 1 |
|  |  | H | Thr143 | Met10 | -2.60 | 2.90 | -b | 1 |
|  |  | IH | Lys146 | Met10 | -15.44 | 2.85 | -b | 3 |
|  |  | H | Trp147 | Met10 | -3.10 | 2.81 | -b | 1 |
|  |  | H | Gln155 | Leu6 | -3.90 | 2.81 | -b | 1 |
| NVDSTDELM | -10.5902 | IH | Arg65 | Met9 | -36.97 | 3.02 | -- | 7 |
|  |  | H | Lys66 | Leu8 | -1.80 | 2.96 | -b | 1 |
|  |  | IH | Lys66 | Met9 | -15.85 | 2.73 | -- | 2 |
|  |  | H | Thr73 | Asp3 | -2.00 | 2.95 | -b | 1 |
|  |  | IH | Asp77 | Asn1 | -33.20 | 2.72 | -b | 4 |
|  |  | H | Asp77 | Val2 | -8.10 | 2.88 | -b | 1 |
|  |  | H | Arg97 | Thr5 | -2.80 | 2.77 | -b | 1 |
|  |  | A | Tyr116 | Asn1 | -0.70 | 4.46 | -b | 1 |
|  |  | H | Thr143 | Asn1 | -0.90 | 2.75 | -- | 1 |
|  |  | H | Lys146 | Asn1 | -6.10 | 2.88 | -* | 2 |
|  |  | H | Trp147 | Asn1 | -1.30 | 3.05 | -b | 1 |
|  |  | H | Gln155 | Glu7 | -6.40 | 2.76 | -- | 1 |
|  |  | H | Leu156 | Asp6 | -0.50 | 3.49 | b- | 1 |
|  |  | A | Tyr159 | Leu8 | -0.50 | 3.60 | -b | 1 |
|  |  | H | Trp167 | Leu8 | -4.60 | 2.87 | -b | 1 |
| WAIIPLSASV | -10.3044 | A | His70 | Ile4 | -0.50 | 3.65 | -- | 1 |
|  |  | H | Tyr84 | Val10 | -2.60 | 2.70 | -b | 1 |
|  |  | H | Arg97 | Ile3 | -7.60 | 2.79 | -b | 1 |
|  |  | H | Thr143 | Val10 | -1.70 | 2.65 | -b | 1 |
|  |  | IH | Lys146 | Val10 | -13.01 | 3.01 | -b | 3 |
|  |  | H | Trp147 | Val10 | -4.90 | 2.80 | -b | 1 |
|  |  | H | Gln155 | Ala2 | -1.30 | 3.00 | -b | 1 |

**Table S6.** Molecular docking calculations and interaction details between selected CTL epitopes and HLA-B*15:02 allele (MOE2020). H, hydrogen bond; IH, Ionic interaction; A, Arene interaction

| **Epitope No.** | **Docking score (kcal/mol)**  **Receptor:**  **HLA-B*15:02** | **Bond Type** | **6VB2** | **Epitope Residues** | **Energy (Kcal/mol)** | **Distance(Å)** | **Backbone Interaction** | **Bond Frequency** |
| --- | --- | --- | --- | --- | --- | --- | --- | --- |
| AIIDIEPDL | -9.7969 | IH | Arg62 | Asp8 | -24.78 | 3.02 | -- | 6 |
|  |  | H | Asn63 | Asp8 | -4.50 | 2.82 | -- | 1 |
|  |  | H | Thr73 | Ala1 | -7.20 | 2.84 | -b | 1 |
|  |  | H | Thr73 | Ile2 | -1.10 | 2.56 | -b | 1 |
|  |  | I | Glu76 | Ala1 | -7.57 | 2.62 | -b | 1 |
|  |  | IH | Arg97 | Asp4 | -23.20 | 3.01 | -- | 4 |
|  |  | H | Glu166 | Leu9 | -4.00 | 2.50 | -b | 1 |
|  |  | H | Trp167 | Asp8 | -3.80 | 2.71 | -- | 1 |
| IHLEWLLGF | -11.2449 | IH | Arg62 | Phe9 | -29.10 | 2.97 | -- | 7 |
|  |  | H | Asn63 | Phe9 | -7.80 | 2.96 | -* | 2 |
|  |  | H | Asn70 | Leu6 | -8.30 | 2.86 | -b | 2 |
|  |  | A | Tyr74 | Trp5 | -0.70 | 4.69 | -- | 1 |
|  |  | IH | Lys146 | Glu4 | -14.54 | 3.07 | -- | 3 |
|  |  | H | Trp147 | Glu4 | -6.40 | 2.72 | -- | 1 |
|  |  | H | Ala149 | Ile1 | -7.10 | 2.78 | bb | 1 |
|  |  | H | Ala149 | His2 | -2.20 | 3.04 | bb | 1 |
|  |  | H | Glu152 | Trp5 | -7.20 | 2.84 | -- | 2 |
|  |  | H | Trp167 | Phe9 | -4.10 | 2.76 | -- | 1 |
| LQKFSFKIA | -11.8487 | H | Arg62 | Gln2 | -5.60 | 2.98 | -- | 2 |
|  |  | H | Asn63 | Leu1 | -10.10 | 2.81 | -b | 1 |
|  |  | H | Asn63 | Gln2 | -6.00 | 2.87 | -* | 2 |
|  |  | H | Asn70 | Ser5 | -1.80 | 3.03 | -b | 1 |
|  |  | H | Thr73 | Ile8 | -1.60 | 2.65 | -b | 1 |
|  |  | H | Tyr74 | Ser5 | -1.80 | 2.76 | -- | 1 |
|  |  | H | Glu76 | Ala9 | -12.60 | 2.65 | -b | 2 |
|  |  | H | Arg79 | Ala9 | -0.80 | 3.58 | -- | 1 |
|  |  | IH | Glu152 | Lys7 | -35.31 | 2.69 | -- | 4 |
| RTVIHLEWL | -11.8487 | H | Asn70 | His5 | -2.70 | 2.81 | -b | 1 |
|  |  | H | Tyr74 | Glu7 | -5.30 | 2.72 | -- | 1 |
|  |  | H | Ser77 | Leu9 | -1.80 | 3.01 | -b | 1 |
|  |  | H | Asn80 | Leu9 | -7.80 | 2.77 | -b | 1 |
|  |  | H | Tyr84 | Leu9 | -4.90 | 2.68 | -b | 1 |
|  |  | IH | Arg97 | Glu7 | -36.25 | 2.80 | -- | 6 |
|  |  | H | Thr143 | Leu9 | -3.30 | 2.69 | -b | 1 |
|  |  | H | Lys146 | Trp8 | -1.10 | 3.21 | b- | 1 |
|  |  | IH | Lys146 | Leu9 | -14.50 | 2.75 | -b | 2 |
|  |  | H | Trp147 | Trp8 | -1.50 | 2.86 | -b | 1 |
|  |  | H | Gln155 | Thr2 | -2.20 | 2.78 | b- | 1 |
|  |  | H | Ala158 | Arg1 | -15.20 | 2.83 | bb | 1 |
|  |  | H | Ala158 | Thr2 | -1.30 | 3.09 | bb | 1 |
|  |  | IH | Glu166 | Arg1 | -38.22 | 2.69 | -b | 4 |
| SLKDVLVSV | -11.3772 | A | Tyr7 | Lys3 | -1.20 | 3.72 | -- | 1 |
|  |  | H | Met45 | Lys3 | -0.60 | 3.32 | -- | 1 |
|  |  | H | Asn63 | Lys3 | -10.30 | 2.88 | -- | 1 |
|  |  | H | Ser67 | Lys3 | -5.70 | 2.88 | -- | 1 |
|  |  | H | Arg97 | Ser8 | -2.00 | 2.87 | -b | 1 |
|  |  | H | Tyr99 | Asp4 | -5.70 | 2.63 | -- | 1 |
|  |  | H | Ser116 | Val9 | -0.60 | 3.34 | -b | 1 |
|  |  | H | Thr143 | Val9 | -3.50 | 2.74 | -b | 1 |
|  |  | H | Lys146 | Ser8 | -6.30 | 2.89 | -- | 1 |
|  |  | H | Trp147 | Ser8 | -1.30 | 2.99 | -b | 1 |
|  |  | H | Glu152 | Val7 | -4.70 | 2.82 | -b | 1 |
|  |  | H | Glu152 | Ser8 | -4.60 | 3.03 | -b | 1 |
|  |  | H | Tyr159 | Leu2 | -1.60 | 2.77 | -b | 1 |
|  |  | A | Trp167 | Leu2 | -0.50 | 4.55 | -- | 1 |
| WKVLSIMAF | -12.5573 | A | Arg62 | Trp1 | -1.20 | 4.38 | -- | 2 |
|  |  | H | Asn63 | Trp1 | -2.60 | 2.99 | -b | 1 |
|  |  | H | Ser77 | Phe9 | -2.30 | 3.01 | -b | 1 |
|  |  | H | Asn80 | Phe9 | -8.50 | 2.79 | -b | 1 |
|  |  | H | Tyr84 | Phe9 | -4.90 | 2.66 | -b | 1 |
|  |  | H | Arg97 | Ile6 | -2.70 | 2.78 | -b | 1 |
|  |  | H | Arg97 | Met7 | -0.70 | 3.23 | -b | 1 |
|  |  | A | Ser116 | Phe9 | -0.60 | 4.04 | -- | 1 |
|  |  | H | Thr143 | Phe9 | -3.30 | 2.65 | -b | 1 |
|  |  | IH | Lys146 | Phe9 | -13.16 | 2.75 | -b | 2 |
|  |  | H | Trp147 | Met7 | -0.50 | 4.35 | b- | 1 |
|  |  | IH | Glu152 | Lys2 | -17.58 | 2.69 | -- | 2 |
|  |  | H | Glu152 | Ser5 | -3.20 | 2.58 | -- | 1 |
|  |  | H | Tyr159 | Trp1 | -2.20 | 2.90 | -b | 1 |
|  |  | A | Tyr159 | Lys2 | -0.50 | 4.26 | -- | 1 |
| ITVGMLIYSM | -10.5191 | H | Tyr9 | Met5 | -2.80 | 2.64 | -b | 1 |
|  |  | IH | Arg62 | Met10 | -22.88 | 3.03 | -b | 6 |
|  |  | H | Asn70 | Met5 | -1.90 | 2.93 | -b | 1 |
|  |  | H | Tyr74 | Gly4 | -3.30 | 2.81 | -b | 1 |
|  |  | A | Trp147 | Ile1 | -0.90 | 4.11 | -b | 1 |
|  |  | IH | Glu152 | Ile1 | -29.80 | 2.81 | -b | 4 |
|  |  | H | Glu152 | Thr2 | -0.50 | 2.86 | -b | 1 |
|  |  | H | Glu166 | Tyr8 | -5.20 | 2.82 | -- | 1 |
|  |  | H | Trp167 | Met10 | -5.50 | 2.75 | -b | 1 |
| KLSSYHVVSV | -10.8728 | H | Arg62 | Lys1 | -5.30 | 3.00 | -b | 1 |
|  |  | H | Asn63 | Lys1 | -0.70 | 3.43 | -b | 1 |
|  |  | H | Glu76 | Ser9 | -3.70 | 2.64 | -- | 1 |
|  |  | H | Ser77 | Val10 | -2.30 | 3.02 | -b | 1 |
|  |  | H | Asn80 | Val10 | -8.50 | 2.80 | -b | 1 |
|  |  | H | Tyr84 | Val10 | -5.00 | 2.68 | -b | 1 |
|  |  | H | Arg97 | Val8 | -0.60 | 2.92 | -b | 1 |
|  |  | H | Thr143 | Val10 | -3.30 | 2.64 | -b | 1 |
|  |  | H | Lys146 | Ser9 | -1.00 | 3.09 | -- | 1 |
|  |  | IH | Lys146 | Val10 | -12.64 | 2.74 | -b | 2 |
|  |  | H | Glu152 | Val8 | -6.80 | 2.97 | -b | 2 |
|  |  | A | Tyr159 | Tyr5 | -0.60 | 4.46 | -- | 1 |
| NVDSTDELM | -9.53267 | H | Asn70 | Asn1 | -3.60 | 2.82 | -- | 1 |
|  |  | H | Tyr74 | Asp3 | -2.80 | 2.62 | -- | 1 |
|  |  | H | Asn80 | Met9 | -6.30 | 2.74 | -b | 1 |
|  |  | H | Tyr84 | Met9 | -4.10 | 2.86 | -b | 1 |
|  |  | IH | Arg97 | Asp3 | -20.25 | 3.05 | -- | 4 |
|  |  | AH | Tyr99 | Asn1 | -3.50 | 3.12 | -- | 2 |
|  |  | IH | Asp114 | Asn1 | -27.13 | 2.76 | -b | 2 |
|  |  | H | Thr143 | Met9 | -3.30 | 2.69 | -b | 1 |
|  |  | IH | Lys146 | Met9 | -15.30 | 2.77 | -b | 2 |
|  |  | IH | Glu152 | Asn1 | -17.36 | 2.71 | -b | 2 |
|  |  | H | Glu152 | Asp3 | -4.80 | 2.87 | -b | 1 |
| WAIIPLSASV | -10.7617 | IH | Glu58 | Trp1 | -16.82 | 2.81 | -b | 3 |
|  |  | H | Arg62 | Ala2 | -5.80 | 2.88 | -b | 1 |
|  |  | H | Asn70 | Ser7 | -4.00 | 2.88 | -* | 2 |
|  |  | H | Tyr74 | Ser7 | -2.60 | 2.68 | -b | 1 |
|  |  | H | Glu76 | Ser9 | -1.20 | 2.57 | -- | 1 |
|  |  | H | Ser77 | Ser9 | -1.70 | 2.67 | -- | 1 |
|  |  | H | Arg97 | Ser7 | -2.90 | 2.82 | -b | 1 |
|  |  | H | Lys146 | Ser9 | -7.70 | 2.81 | -b | 1 |
|  |  | H | Trp167 | Trp1 | -2.30 | 2.85 | -b | 1 |

**Table S7.** Atom-atom interactions across TLR3-MPXV-1 interface obtained using PDBsum server

| TLR3 residues | | | | | MPXV-1 vaccine construct | | | | |  |
| --- | --- | --- | --- | --- | --- | --- | --- | --- | --- | --- |
| Atom no. | **Atom name** | **Residue name** | **Residue no.** | **Chain** | **Atom no.** | **Atom name** | **Residue name** | **Residue no.** | **Chain** | **Distance & Bond Type** |
| 1 | N | Ala | 22 | B | 14434 | O | Gly | 240 | A | 2.8-H |
| 1 | N | Ala | 22 | B | 14482 | O | Pro | 244 | A | 2.9-H |
| 1 | N | Ala | 22 | B | 14493 | O | Gly | 245 | A | 2.88-H |
| 1 | N | Ala | 22 | B | 14500 | OG | Ser | 246 | A | 2.73-H |
| 29 | OG | Ser | 24 | B | 14445 | OH | Tyr | 241 | A | 2.76-H |
| 145 | NE2 | His | 32 | B | 14178 | OD2 | Asp | 220 | A | 2.74-H |
| 235 | NE2 | His | 39 | B | 15137 | OD1 | Asp | 291 | A | 2.81-H |
| 556 | OG1 | Thr | 59 | B | 15138 | OD2 | Asp | 291 | A | 2.69-H |
| 574 | NE2 | His | 60 | B | 15138 | OD2 | Asp | 291 | A | 2.89-H |
| 2435 | OE1 | Glu | 175 | B | 14818 | ND2 | Asn | 269 | A | 2.91-H |
| 2854 | NZ | Lys | 201 | B | 14833 | OE2 | Glu | 270 | A | 2.69-H |
| 4139 | OH | Tyr | 283 | B | 14935 | OD1 | Asp | 277 | A | 2.61-H |
| 4530 | OH | Tyr | 307 | B | 14923 | OD1 | Asp | 276 | A | 2.57-H |
| 4916 | NZ | Lys | 330 | B | 14969 | OD2 | Asp | 280 | A | 2.74-H |
| 4939 | NH1 | Arg | 331 | B | 14968 | OD1 | Asp | 280 | A | 2.7-H |
| 5403 | NE2 | His | 359 | B | 13105 | O | Leu | 145 | A | 2.91-H |
| 5470 | OE2 | Glu | 363 | B | 13232 | NZ | Lys | 153 | A | 2.7-H |
| 5783 | OH | Tyr | 383 | B | 13175 | O | Met | 149 | A | 2.66-H |
| 5817 | OG | Ser | 385 | B | 13232 | NZ | Lys | 153 | A | 2.87-H |
| 6646 | OD1 | Asp | 437 | B | 13296 | OH | Tyr | 157 | A | 2.74-H |
| 7043 | OH | Tyr | 462 | B | 13316 | NE2 | His | 158 | A | 3.01-H |
| 7414 | NH1 | Arg | 484 | B | 11933 | O | Leu | 72 | A | 2.89-H |
| 7415 | NH2 | Arg | 484 | B | 11933 | O | Leu | 72 | A | 2.94-H |
| 7494 | NH2 | Arg | 488 | B | 13364 | O | Ser | 161 | A | 2.73-H |
| 7494 | NH2 | Arg | 488 | B | 13400 | O | Ala | 164 | A | 2.71-H |
| 7930 | ND2 | Asn | 515 | B | 13400 | O | Ala | 164 | A | 3.03-H |
| 8308 | NE2 | His | 539 | B | 13417 | O | Tyr | 165 | A | 2.82-H |
| 8815 | OE1 | Glu | 570 | B | 13413 | OH | Tyr | 165 | A | 2.59-H |
| 8828 | OG | Ser | 571 | B | 13413 | OH | Tyr | 165 | A | 2.87-H |
| 9176 | OD2 | Asp | 592 | B | 11846 | N | Gly | 66 | A | 2.79-H |
| 9592 | NZ | Lys | 619 | B | 13431 | OD1 | Asn | 166 | A | 2.92-H |
| 145 | NE2 | His | 32 | B | 14178 | OD2 | Asp | 220 | A | 2.74-salt bridge |
| 235 | NE2 | His | 39 | B | 15138 | OD2 | Asp | 291 | A | 2.81-salt bridge |
| 574 | NE2 | His | 60 | B | 15137 | OD1 | Asp | 291 | A | 2.89-salt bridge |
| 2104 | OD2 | Asp | 153 | B | 14785 | NH1 | Arg | 267 | A | 2.71-salt bridge |
| 2854 | NZ | Lys | 201 | B | 14833 | OE2 | Glu | 270 | A | 2.69-salt bridge |
| 4916 | NZ | Lys | 330 | B | 14969 | OD2 | Asp | 280 | A | 2.74-salt bridge |
| MPXV-1 residues in non-bonded contacts with TLR3: Pro237, Gly240, Tyr241, Pro244, Gly245, Ser 246, Pro247, Tyr241, Asp235, Gly236, Pro263, Gly264, Asp220, Asp235, Pro237, Asp291, Gly264, Val266, Arg267, Phe272, Glu270, Asp277, Ile133, Ala138, Val142, Tyr139, Leu 145, Asp280, Val142, Trp84, Val142, Ile146, Leu145, Met149, Lys153, Trp84, Met149, Lys153, Tyr157, Val76, His158, Ser73, Ser161, Ser161, Leu72, Ser73, Ser161, Ala164, Ala68, Tyr165 | | | | | | | | | | |

**Table S8.** Atom-atom interactions across TLR3-MPXV-2 interface obtained using PDBsum server

| TLR3 residues | | | | | MPXV-2 vaccine construct | | | | |  |
| --- | --- | --- | --- | --- | --- | --- | --- | --- | --- | --- |
| Atom no. | **Atom name** | **Residue name** | **Residue no.** | **Chain** | **Atom no.** | **Atom name** | **Residue name** | **Residue no.** | **Chain** | **Distance & Bond Type** |
| 24 | N | Ser | 24 | A | 15038 | O | Gly | 286 | B | 3.02-H |
| 27 | O | Ser | 24 | A | 15110 | NE2 | Gln | 291 | B | 2.76-H |
| 145 | NE2 | His | 32 | A | 14980 | OE2 | Glu | 282 | B | 2.73-H |
| 219 | O | Ser | 38 | A | 15110 | NE2 | Gln | 291 | B | 2.75-H |
| 271 | NZ | Lys | 41 | A | 15094 | OE1 | Glu | 290 | B | 2.72-H |
| 2419 | NE2 | Gln | 174 | A | 11318 | OE2 | Glu | 33 | B | 2.75-H |
| 2419 | NE2 | Gln | 174 | A | 11333 | O | Val | 34 | B | 2.81-H |
| 2832 | NZ | Lys | 200 | A | 15511 | OD1 | Asp | 320 | B | 2.74-H |
| 2854 | NZ | Lys | 201 | A | 15486 | O | Glu | 318 | B | 2.76-H |
| 3300 | ND2 | Asn | 229 | A | 15484 | OE2 | Glu | 3158 | B | 2.76-H |
| 3636 | NH1 | Arg | 251 | A | 11357 | O | Ala | 36 | B | 2.92-H |
| 3637 | NH2 | Arg | 251 | A | 11357 | O | Ala | 36 | B | 3.12-H |
| 3637 | NH2 | Arg | 251 | A | 15512 | OD2 | Asp | 320 | B | 6 |
| 3658 | ND2 | Asn | 252 | A | 15514 | O | Asp | 320 | B | 2.82-H |
| 3689 | OG | Ser | 254 | A | 15819 | NZ | Lys | 344 | B | 3.01-H |
| 4093 | OD2 | Asp | 280 | A | 15819 | NZ | Lys | 344 | B | 2.84-H |
| 4362 | O | Pro | 298 | A | 10860 | NZ | Lys | 3 | B | 2.73-H |
| 4381 | OE1 | Gln | 299 | A | 10860 | NZ | Lys | 3 | B | 2.73-H |
| 4511 | OE1 | Glu | 306 | A | 15805 | OG | Ser | 343 | B | 2.62-H |
| 4846 | OH | Tyr | 326 | A | 15802 | N | Ser | 343 | B | 3.02-H |
| 4882 | ND2 | Asn | 328 | A | 15807 | O | Ser | 343 | B | 2.75-H |
| 4916 | NZ | Lys | 330 | A | 15965 | OE1 | Glu | 355 | B | 2.81-H |
| 4916 | NZ | Lys | 330 | A | 15966 | OE2 | Glu | 355 | B | 2.7-H |
| 5388 | OE2 | Glu | 358 | A | 15761 | ND2 | Asn | 339 | B | 2.81-H |
| 5403 | NE2 | His | 359 | A | 16252 | O | Lys | 375 | B | 2.95-H |
| 5783 | OH | Tyr | 383 | A | 16238 | O | Ser | 374 | B | 2.75-H |
| 6202 | NE2 | His | 410 | A | 12962 | O | Ala | 148 | B | 2.76-H |
| 6598 | OE2 | Glu | 434 | A | 12975 | N | Gly | 150 | B | 2.85-H |
| 7414 | NH1 | Arg | 484 | A | 16104 | O | Asp | 365 | B | 2.92-H |
| 7415 | NH2 | Arg | 484 | A | 16104 | O | Asp | 365 | B | 2.74-H |
| 145 | NE2 | His | 32 | A | 14980 | OE2 | Glu | 282 | B | 2.73-salt bridges |
| 234 | ND1 | His | 39 | A | 15095 | OE2 | Glu | 290 | B | 3.66-salt bridges |
| 271 | NZ | Lys | 41 | A | 15094 | OE1 | Glu | 290 | B | 2.72-salt bridges |
| 2832 | NZ | Lys | 200 | A | 15511 | OD1 | Asp | 320 | B | 2.74-salt bridges |
| 3637 | NH2 | Arg | 251 | A | 15512 | OD2 | Asp | 320 | B | 2.72-salt bridges |
| 4093 | OD2 | Asp | 280 | A | 15819 | NZ | Lys | 344 | B | 2.84-salt bridges |
| MPXV-2 residues in non-bonded contacts with TLR3: Lys3, Thr28, Phe29, Glu30, Val31, Ala33, Ala34, Ala145, Gly146, Tyr247, Asn248, Glu254, Glu279, Ser282, Gly 283, Leu284, Thr 285, Glu287, Gln288, Tyr 291, Met295, Phe296, Glu315, Thr316, Asp317, Asn336, Gly338, Ser339, Ser340, Lys341, Gly342, Glu352, Leu 367, Gly375, Ser377 | | | | | | | | | | |

**Table S9.** Atom-atom interactions across TLR2-MPXV-3 interface obtained using PDBsum server

| TLR2 | | | | | MPXV-3 | | | | |  |
| --- | --- | --- | --- | --- | --- | --- | --- | --- | --- | --- |
| Atom no. | **Atom name** | **Residue name** | **Residue no.** | **Chain** | **Atom no.** | **Atom name** | **Residue name** | **Residue no.** | **Chain** | **Distance & Bond Type** |
| 4590 | NE2 | His | 318 | A | 11744 | O | Leu | 193 | B | 2.97-H |
| 4640 | NH1 | Arg | 321 | A | 9710 | OE1 | Gln | 58 | B | 2.81-H |
| 4640 | NH1 | Arg | 321 | A | 11761 | OE1 | Glu | 194 | B | 2.71-H |
| 4641 | NH2 | Arg | 321 | A | 9653 | OG1 | Thr | 55 | B | 2.87-H |
| 4678 | O | Tyr | 323 | A | 11687 | OH | Tyr | 190 | B | 2.74-H |
| 5094 | NZ | Lys | 347 | A | 11850 | O | Phe | 199 | B | 2.77-H |
| 6712 | NH1 | Arg | 447 | A | 12566 | OG | Ser | 243 | B | 2.81-H |
| 6713 | NH2 | Arg | 447 | A | 12568 | O | Ser | 243 | B | 2.93-H |
| 6713 | NH2 | Arg | 447 | A | 12614 | O | Ala | 246 | B | 2.88-H |
| 7344 | NH1 | Arg | 486 | A | 12217 | OE2 | Glu | 221 | B | 2.74-H |
| 7345 | NH2 | Arg | 486 | A | 12146 | O | Val | 217 | B | 2.82-H |
| 7345 | NH2 | Arg | 486 | A | 12217 | OE2 | Glu | 221 | B | 2.76-H |
| 7712 | NE | Arg | 508 | A | 12380 | O | Val | 231 | B | 2.97-H |
| 7714 | NH1 | Arg | 508 | A | 12216 | OE1 | Glu | 221 | B | 2.69-H |
| 7715 | NH2 | Arg | 508 | A | 12184 | O | His | 219 | B | 2.96-H |
| 7715 | NH2 | Arg | 508 | A | 12199 | O | Leu | 220 | B | 2.82-H |
| 8106 | ND1 | His | 531 | A | 12216 | OE1 | Glu | 221 | B | 2.81-H |
| 8122 | OG1 | Thr | 532 | A | 12199 | O | Leu | 220 | B | 3.23-H |
| 8581 | NZ | Lys | 561 | A | 12273 | O | Ala | 224 | B | 2.65-H |
| 8608 | O | Ser | 563 | A | 12346 | NZ | Lys | 229 | B | 2.83-H |
| 8619 | O | Gly | 564 | A | 12346 | NZ | Lys | 229 | B | 2.8-H |
| 8649 | NZ | Lys | 567 | A | 12283 | O | Ala | 225 | B | 2.79-H |
| 4640 | NH1 | Arg | 321 | A | 11762 | OE2 | Glu | 194 | B | 2.71-salt bridges |
| 7344 | NH1 | Arg | 486 | A | 12216 | OE1 | Glu | 221 | B | 2.74-salt bridges |
| 7714 | NH1 | Arg | 508 | A | 12217 | OE2 | Glu | 221 | B | 2.69-salt bridges |
| 8106 | ND1 | His | 531 | A | 12217 | OE2 | Glu | 221 | B | 2.81-salt bridges |
| MPXV-3 residues in non-bonded contact with TLR2: Thr55, Gln58, Tyr190, Leu193, Glu194, Leu196, Leu197, Phe199, Leu203, Gln204, Tyr 214, Val217, His219, Leu220, Glu221, Ala224, Ala225, Tyr226, Leu228, Lys229, Val231, Leu232, Val235, Trp239, Leu242, Ser243, Met245, Ala249, Val253, Leu256, Met260 | | | | | | | | | | |

**Table S10.** Hydrogen bonds at the TLR3– MPXV-1 construct interface (with occupancy ≥ 2%)

| **TLR3 residue-atom** | **MPXV-1 residue-atom** | **Occupancy (%)** | **Bond distance** |
| --- | --- | --- | --- |
| His338-NE2 | Leu145-O | 52.4 | 2.86 |
| Glu549-OE1 | Tyr165-OH | 33.6 | 2.69 |
| Lys180-NZ | Glu270-OE2 | 26.4 | 2.81 |
| Glu342-OE2 | Lys153-NZ | 18.4 | 2.8 |
| Arg310-NH2 | Asp280-OD2 | 15.4 | 2.81 |
| Asp416-OD1 | Tyr157-OH | 12.6 | 2.75 |
| Ala1-N | Ser246-OG | 9.22 | 2.86 |
| Tyr286-OH | Asp276-OD1 | 8.3 | 2.69 |
| Arg463-NH2 | Leu72-O | 8.05 | 2.87 |
| His39-NE2 | Asp291-OD1 | 7.95 | 2.84 |
| His108-ND1 | Arg267-NH1 | 7.52 | 2.89 |
| His18-NE2 | Asp291-OD2 | 7.43 | 2.83 |
| Asp132-OD2 | Arg267-NH1 | 6.75 | 2.78 |
| Lys309-NZ | Asp280-OD2 | 6.12 | 2.81 |
| Tyr444-OH | Ser161-OG | 5.05 | 2.84 |
| Arg467-NE | Ser161-O | 3.39 | 2.87 |
| Ser550-OG | Tyr165-OH | 2.81 | 2.83 |
| Ser3-OG | Tyr241-OH | 2.75 | 2.83 |
| Tyr262-OH | Asp277-OD1 | 2.55 | 2.68 |

**Table S11**. Hydrogen bonds at the TLR3– MPXV-2 construct interface (with occupancy ≥ 2%)

| **TLR3 residue-atom** | **MPXV-1 residue-atom** | **Occupancy (%)** | **Bond distance** |
| --- | --- | --- | --- |
| Lys180-NZ | Gly318-O | 75.3 | 2.81 |
| Asp259-OD2 | Lys344-NZ | 56.2 | 2.78 |
| Arg230-NH2 | Gly320-OD2 | 53.9 | 2.77 |
| Arg201-NH1 | Thr31-O | 42.4 | 2.84 |
| Lys179-NZ | Asp320-OD1 | 36.2 | 2.76 |
| Arg230-NH2 | Thr31-O | 14.1 | 2.83 |
| Glu285OE1 | Ser343-OG | 12.3 | 2.63 |
| Glu413-OE1 | Gly150-N | 11 | 2.86 |
| Ser17-O | Gln291-NE2 | 10.4 | 2.85 |
| Arg230-NH1 | Thr31-O | 8.09 | 2.86 |
| Asn231-ND2 | Asp320-O | 6.93 | 2.87 |
| Tyr305-OH | Ser343-N | 6.84 | 2.91 |
| Gln278-OE1 | Lys3-NZ | 5.46 | 2.82 |
| Lys180-CE | Glu318-O | 4.42 | 2.92 |
| Arg463-NH1 | Asp365-O | 2.83 | 2.83 |
| Arg304-NH1 | Glu257-OE1 | 2.81 | 2.81 |
| Ser3-O | Gln291-NE2 | 2.88 | 2.88 |

**Table S12**. Hydrogen bonds at the TLR2– MPXV-3 construct interface (with occupancy ≥ 2%)

| **TLR2 residue-atom** | **MPXV-3 residue-atom** | **Occupancy (%)** | **Bond distance** |
| --- | --- | --- | --- |
| Arg421-NH1 | Ser243-OG | 23.4 | 2.86 |
| Arg482-NH2 | Glu221-OE2 | 19.9 | 2.81 |
| Arg 482-NH2 | Val217-O | 18.7 | 2.84 |
| Arg 482-NH1 | Glu221-OE2 | 15.5 | 2.78 |
| Lys535-NZ | Ala224-O | 9.18 | 2.83 |
| Asn440-O | Trp239-NE1 | 6.37 | 2.83 |
| Arg295-NH1 | Glu194-OE2 | 4.63 | 2.81 |
| Tyr297-O | Tyr190-OH | 4.06 | 2.81 |
| Arg295-O | Tyr190-CD2 | 2.26 | 2.93 |
| His292-NE2 | Leu193-O | 0.491 | 2.88 |
| Lys541-NZ | Met225-O | 0.464 | 2.82 |
| Lys321-NZ | Phe199-O | 0.3 | 2.84 |
| Arg460-NE | Tyr214-OH | 0.255 | 2.9 |
| Arg460-NH2 | Glu221-OE1 | 0.236 | 2.82 |

**Table S13.** The complementary DNA sequence of the constructed monkeypox vaccines was subjected to in-silico cloning and RNA structure prediction in this study. The N-terminal contains inserted XhoI restriction site, and the C-terminal contains inserted SacI restriction site (shown in bold letters).

| Vaccine | cDNA sequence |
| --- | --- |
| MPXV-1  (927) | **CTCGAG**GGTATCATCAACACCCTGCAGAAATACTACTGCCGTGTTCGTGGTGGTCGTTGCGCTGTTCTGTCTTGCCTGCCGAAAGAAGAACAGATCGGTAAATGCTCTACCCGTGGTCGTAAATGCTGCCGTCGTAAAAAAGAAGCTGCTGCTAAAGCTAAATTCGTTGCTGCTTGGACCCTGAAAGCTGCTGCTGGTGGTGGTTCTGCTATCATCCCGCTGTCTGCTTCTGTTGCTGCTTACATCCACCTGGAATGGCTGCTGGGTTTCGCTGCTTACCTGCAGAAATTCTCTTTCAAAATCGCTGCTGCTTACCGTACCGTTATCCACCTGGAATGGCTGGCTGCTTACTCTCTGAAAGACGTTCTGGTTTCTGTTGCTGCTTACTGGAAAGTTCTGTCTATCATGGCTTTCGCTGCTTACATCACCGTTGGTATGCTGATCTACTCTATGGCTGCTTACAAACTGTCTTCTTACCACGTTGTTTCTGTTGCTGCTTACAACGTTGACTCTACCGACGAACTGATGGCTGCTTACTGGGCTATCATCCCGCTGTCTGCTTCTGTTGGTGGTGGTTCTCTGTCTGCTGCTACCGAAACCTACTCTGGTCTGACCCCGGAACAGAAAGCTTACGTTCCGGCTATGTTCGGTCCGGGTCCGGGTGACTCTGGTTACCACTCTCTGGACCCGAACGCTGTTTGCGAAACCGACGGTCCGGGTCCGGGTTACGGTGCTCCGGGTTCTCCGACCAACCTGGAATTCATCAACACCGGTTCTTCTAAAGGTCCGGGTCCGGGTTGCGTTCGTTCTAACGAAGAATTCGACCCGGTTGACGACGGTCCGGACGACGAAACCGACCTGTCTAAACTGTCTAAAGACGGTGGTGGTTCTACCGGTGCTCTGCTGGCTGCTGGTGCTGCT**GAGCTC** |
| MPXV-2  (1176) | **GTCGAG**ATGGCCAAGCTGAGCACCGACGAGCTGCTGAAGGAGATGACCCTGCTGGAGCTGAGCGACTTCGTGAAGAAGTTCGAGGAGACCTTCGAGGTGACCGCCGCCGCCCCCGTGGCCGTGGCCGCCGCCGGCGCCGCCCCCGCCGGCGCCGCCGTGGAGGCCGCCGAGGAGCAGAGCGAGTTCGACGTGATCCTGGAGGCCGCCGGCGACAAGAAGATCGGCGTGATCAAGGTGGTGAGGGAGATCGTGAGCGGCCTGGGCCTGAAGGAGGCCAAGGACCTGGTGGACGGCGCCCCCAAGCCCCTGCTGGAGAAGGTGGCCAAGGAGGCCGCCGACGAGGCCAAGGCCAAGCTGGAGGCCGCCGGCGCCACCGTGACCGTGAAGGAGGCCGCCGCCAAGGCCAAGTTCGTGGCCGCCTGGACCCTGAAGGCCGCCGCCGGCGGCGGCAGCGCCATCATCCCCCTGAGCGCCAGCGTGGCCGCCTACATCCACCTGGAGTGGCTGCTGGGCTTCGCCGCCTACCTGCAGAAGTTCAGCTTCAAGATCGCCGCCGCCTACAGGACCGTGATCCACCTGGAGTGGCTGGCCGCCTACAGCCTGAAGGACGTGCTGGTGAGCGTGGCCGCCTACTGGAAGGTGCTGAGCATCATGGCCTTCGCCGCCTACATCACCGTGGGCATGCTGATCTACAGCATGGCCGCCTACAAGCTGAGCAGCTACCACGTGGTGAGCGTGGCCGCCTACAACGTGGACAGCACCGACGAGCTGATGGCCGCCTACTGGGCCATCATCCCCCTGAGCGCCAGCGTGGGCGGCGGCAGCCTGAGCGCCGCCACCGAGACCTACAGCGGCCTGACCCCCGAGCAGAAGGCCTACGTGCCCGCCATGTTCGGCCCCGGCCCCGGCGACAGCGGCTACCACAGCCTGGACCCCAACGCCGTGTGCGAGACCGACGGCCCCGGCCCCGGCTACGGCGCCCCCGGCAGCCCCACCAACCTGGAGTTCATCAACACCGGCAGCAGCAAGGGCCCCGGCCCCGGCTGCGTGAGGAGCAACGAGGAGTTCGACCCCGTGGACGACGGCCCCGACGACGAGACCGACCTGAGCAAGCTGAGCAAGGACGGCGGCGGCAGCACCGGCGCCCTGCTGGCCGCCGGCGCCGCCGCC**GAGCTC** |
| MPXV-3  (1260) | **CTCGAG**ATGGCTGAAAACCCGAACATCGACGACCTGCCGCTGGCTGCTCTGGGTGCTGCTGACCTGGCTCTGGCTACCGTTAACGACCTGATCGCTAACCTGCGTGAACGTGCTGAAGAAACCCGTGCTGAAACCCGTACCCGTGTTGAAGAACGTCGTGCTCGTCTGACCAAATTCCAGGAAGACCTGCCGGAACAGTTCATCGAACTGCGTGACAAATTCACCACCGAAGAACTGCGTAAAGCTGCTGAAGGTTACCTGGAAGCTGCTACCAACCGTTACAACGAACTGGTTGAACGTGGTGAAGCTGCTCTGCAGCGTCTGCGTTCTCAGACTGCGTTCGAAGACGCTTCGGCTCGTGCTGAAGGTTACGTTGACCAGGCTGTTGAACTGACCCAGGAAGCTCTGGGTACCGTTGCTTCTCAGACCCGTGCTGTTGGTGAACGTGCTGCTAAACTGGTTGGTATCGAACTGGAAGCTGCTGCTAAAGCTAAATTCGTTGCTGCTTGGACCCTGAAAGCTGCTGCTGGTGGTGGTTCTGCTATCATCCCGCTGTCTGCTTCTGTTGCTGCTTACATCCACCTGGAATGGCTGCTGGGTTTCGCTGCTTACCTGCAGAAATTCTCTTTCAAAATCGCTGCTGCTTACCGTACCGTTATCCACCTGGAATGGCTGGCTGCTTACTCTCTGAAAGACGTTCTGGTTTCTGTTGCTGCTTACTGGAAAGTTCTGTCTATCATGGCTTTCGCTGCTTACATCACCGTTGGTATGCTGATCTACTCTATGGCTGCTTACAAACTGTCTTCTTACCACGTTGTTTCTGTTGCTGCTTACAACGTTGACTCTACCGACGAACTGATGGCTGCTTACTGGGCTATCATCCCGCTGTCTGCTTCTGTTGGTGGTGGTTCTCTGTCTGCTGCTACCGAAACCTACTCTGGTCTGACCCCGGAACAGAAAGCTTACGTTCCGGCTATGTTCGGTCCGGGTCCGGGTGACTCTGGTTACCACTCTCTGGACCCGAACGCTGTTTGCGAAACCGACGGTCCGGGTCCGGGTTACGGTGCTCCGGGTTCTCCGACCAACCTGGAATTCATCAACACCGGTTCTTCTAAAGGTCCGGGTCCGGGTTGCGTTCGTTCTAACGAAGAATTCGACCCGGTTGACGACGGTCCGGACGACGAAACCGACCTGTCTAAACTGTCTAAAGACGGTGGTGGTTCTACCGGTGCTCTGCTGGCTGCTGGTGCTGCT**GAGCTC** |

**Monkeypox Proteins (191)**

**Final Candidates Proteins (8)**

**Figure S1.** Hierarchical representation of Vax-ELAN pipeline followed in selecting best-ranked outer membrane proteins of MPXV. Out of 191 proteins of MPXV, the integrated pipeline finalized eight proteins (A21L, A30L, A43R, B8R, B9R, B20R, C22L, and J2R) as potential vaccine candidates. These proteins can be subjected to novel B-cell and T-cell epitopes mapping.


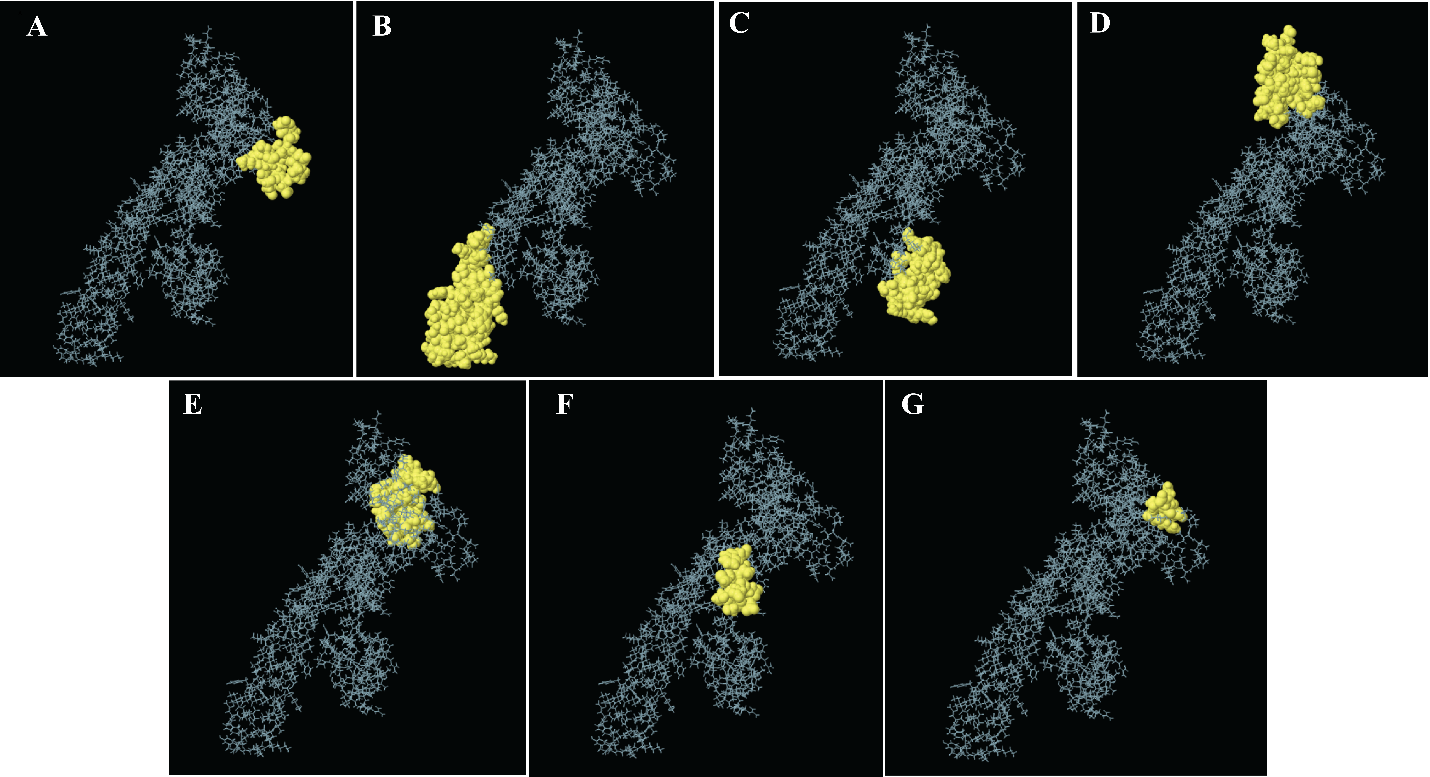


**Figure S2.** Conformational B-cell epitopes predicted in MPXV-1. The light-yellow spheres showing epitopes containing **(A)** 15 residues (AA 273–287) with score 0.769 **(B)** 43 residues (AA 89, 92–134) with score 0.759 **(C)** 32 residues (AA 1–22, 30–39) with score 0.715 **(D)** 31 residues (191–220, 225,226) with score 0.667 **(E)** 36 residues (AA 227–265) with score 0.626 **(F)** 9 residues (AA 167–172, 174, 175, 178) with score 0.621 **(G)** 5 residues (AA267–272) with score 0.552.


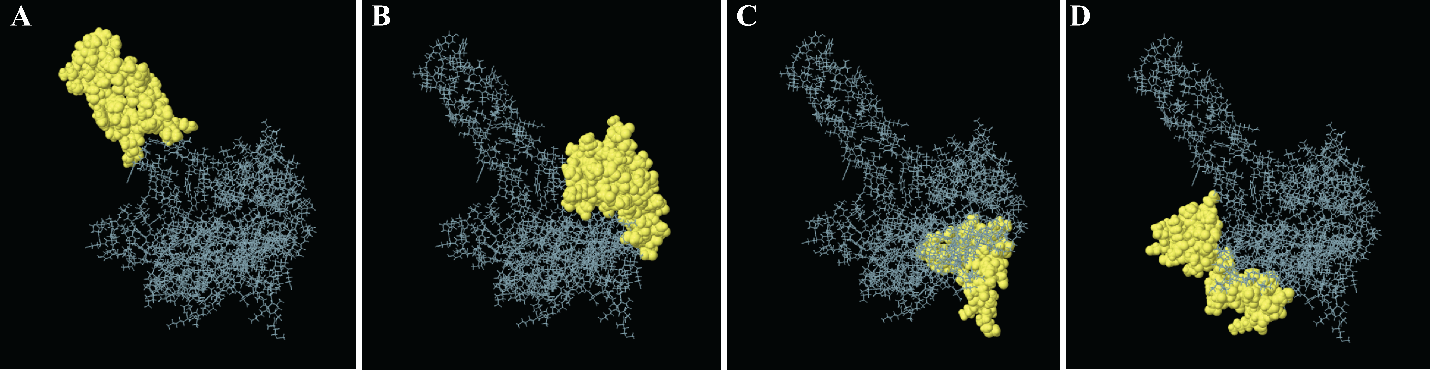


**Figure S3**. Conformational B-cell epitopes predicted in MPXV-2. The light-yellow spheres showing epitopes containing **(A)** 45 residues (AA 174–218) with score 0.865 **(B)** 67 residues (AA 52–102, 114–131) with score 0.704 **(C)** 34 residues (AA 1–35) with score of 0.671 **(D)** 61 residues (AA277–302, 307–319, 335–344, 347–348, 350–353, 371–375, 377) with score 0.628.


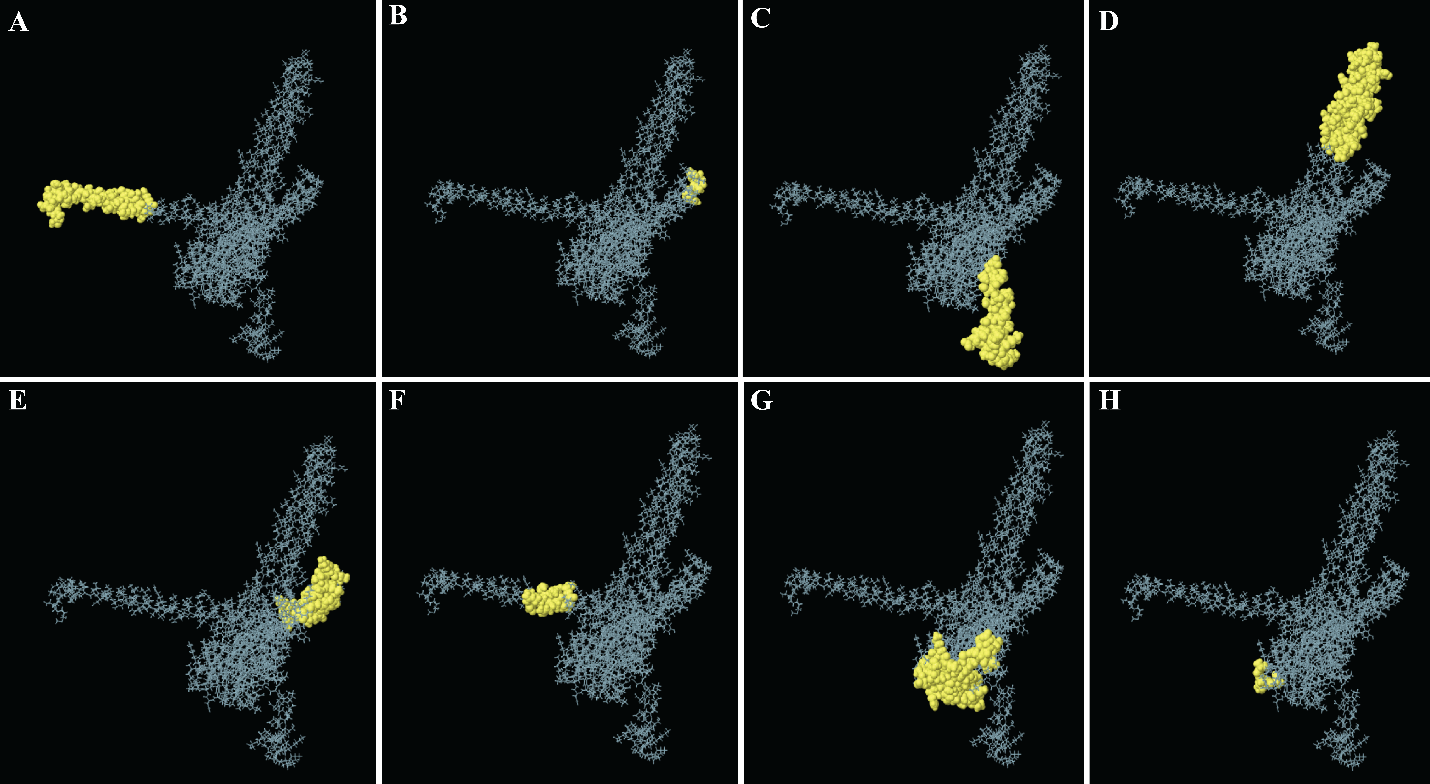


**Figure S4**. Conformational B-cell epitopes predicted in MPXV-3. The light-yellow spheres showing epitopes containing **(A)** 35 residues (AA1-35) with score 0.902 **(B)** 5 residues (AA 222–226) with score 0.835 **(C)** 34 residues (AA384–417) with score 0.828 **(D)** 52 residues (AA 82–134) with score 0.772 **(E)** 29 residues (AA207–-221, 227–239, 242) with score 0.675 **(F)** 11 residues (AA36-46) with score **(G)** 46 residues (AA267, 270–283, 337, 352–383) with score 0.526 **(H)** 6 residues (AA174–179) with score 0.522.


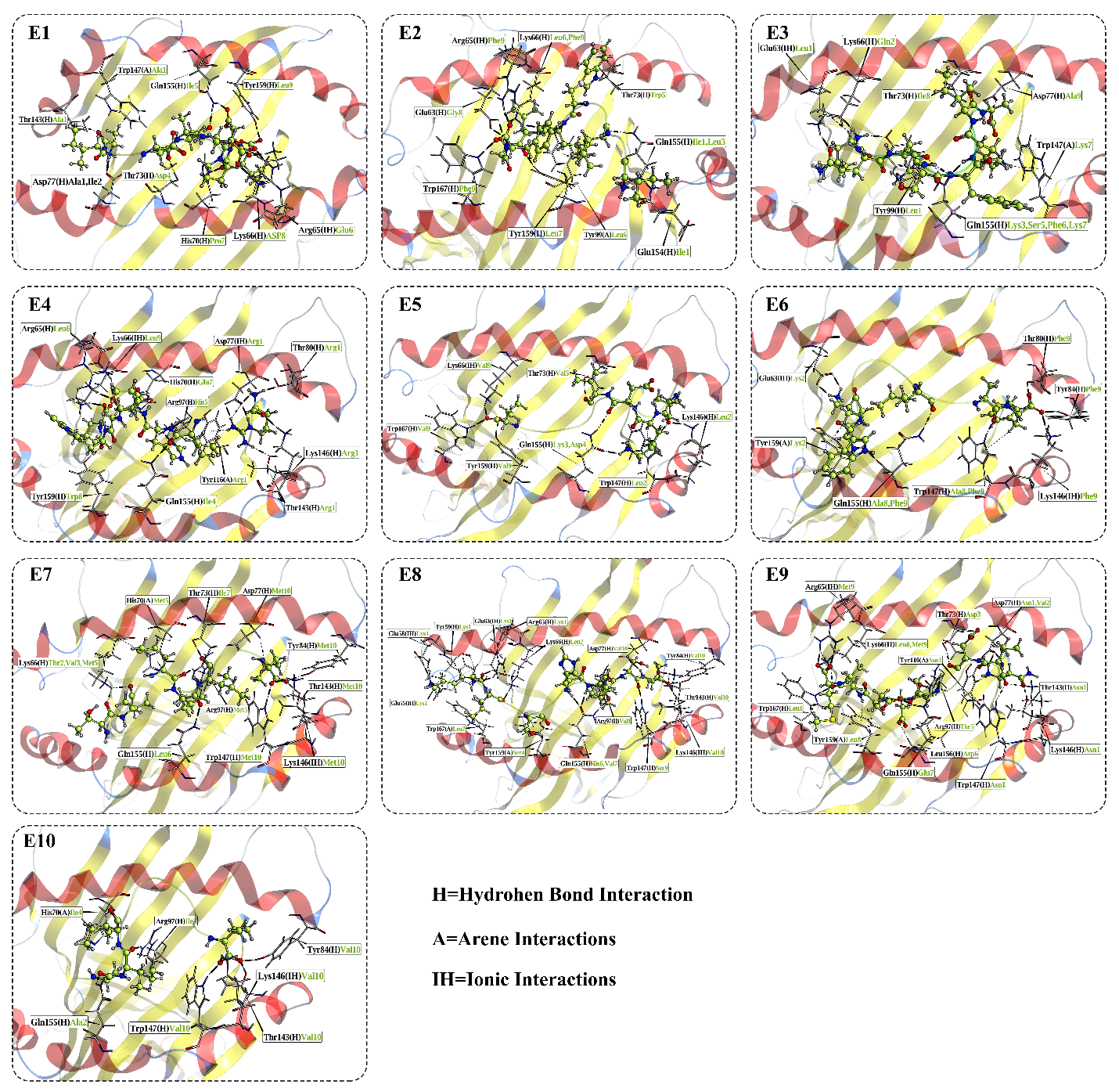


**Figure S5.** Predominant binding mode of selected CTL epitopes with HLA-A*0201 allele (MOE2020). E1, AIIDIEPDL; E2, IHLEWLLGF; E3, LQKFSFKIA; E4, RTVIHLEWL; E5, SLKDVLVSV; E6, WKVLSIMAF; E7, ITVGMLIYSM; E8, KLSSYHVVSV; E9, NVDSTDELM; E10, WAIIPLSASV.


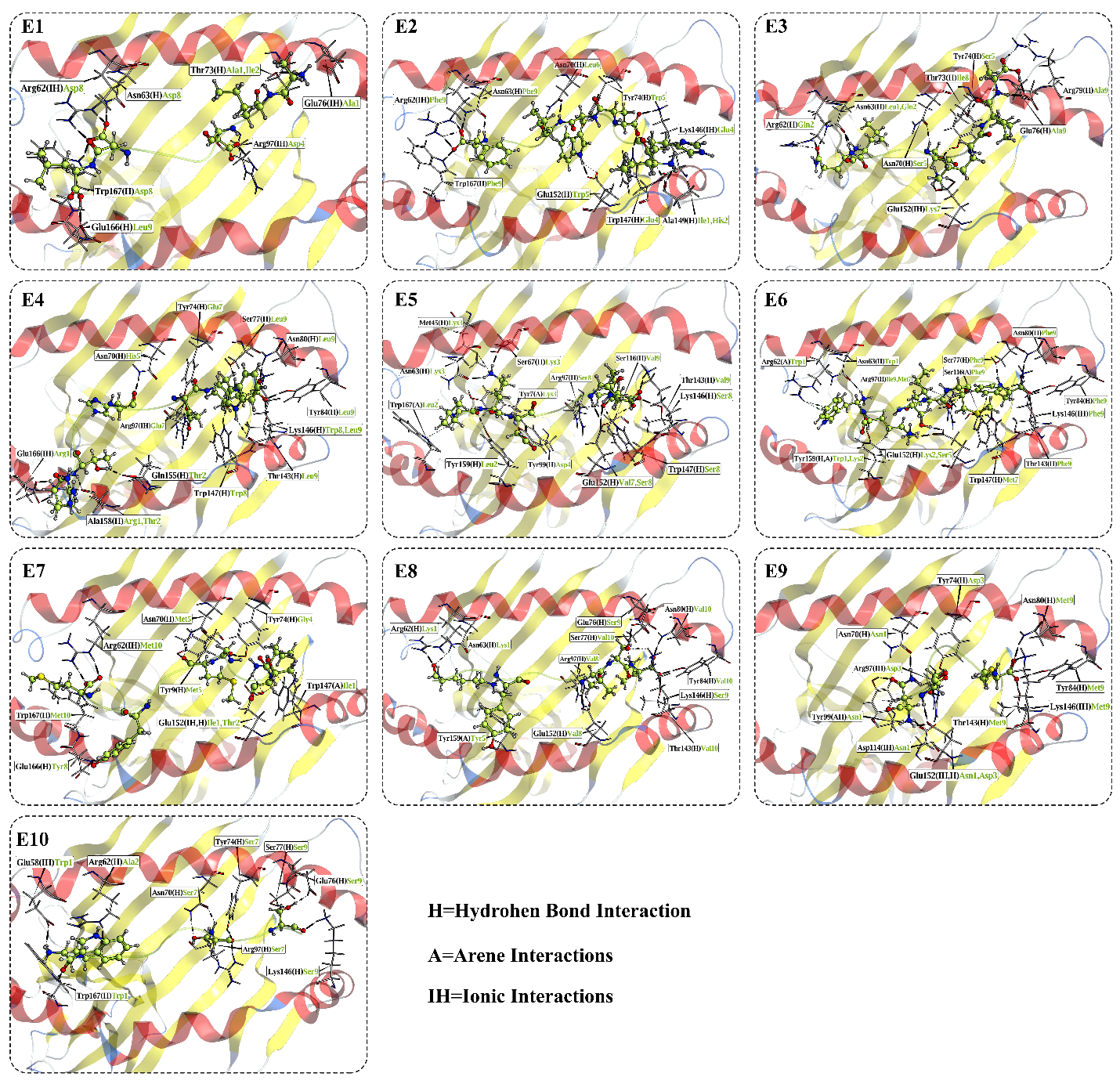


**Figure S6**. Predominant binding mode of selected CTL epitopes with HLA-B*15:02 allele (MOE2020). E1, AIIDIEPDL; E2, IHLEWLLGF; E3, LQKFSFKIA; E4, RTVIHLEWL; E5, SLKDVLVSV; E6, WKVLSIMAF; E7, ITVGMLIYSM; E8, KLSSYHVVSV; E9, NVDSTDELM; E10, WAIIPLSASV.


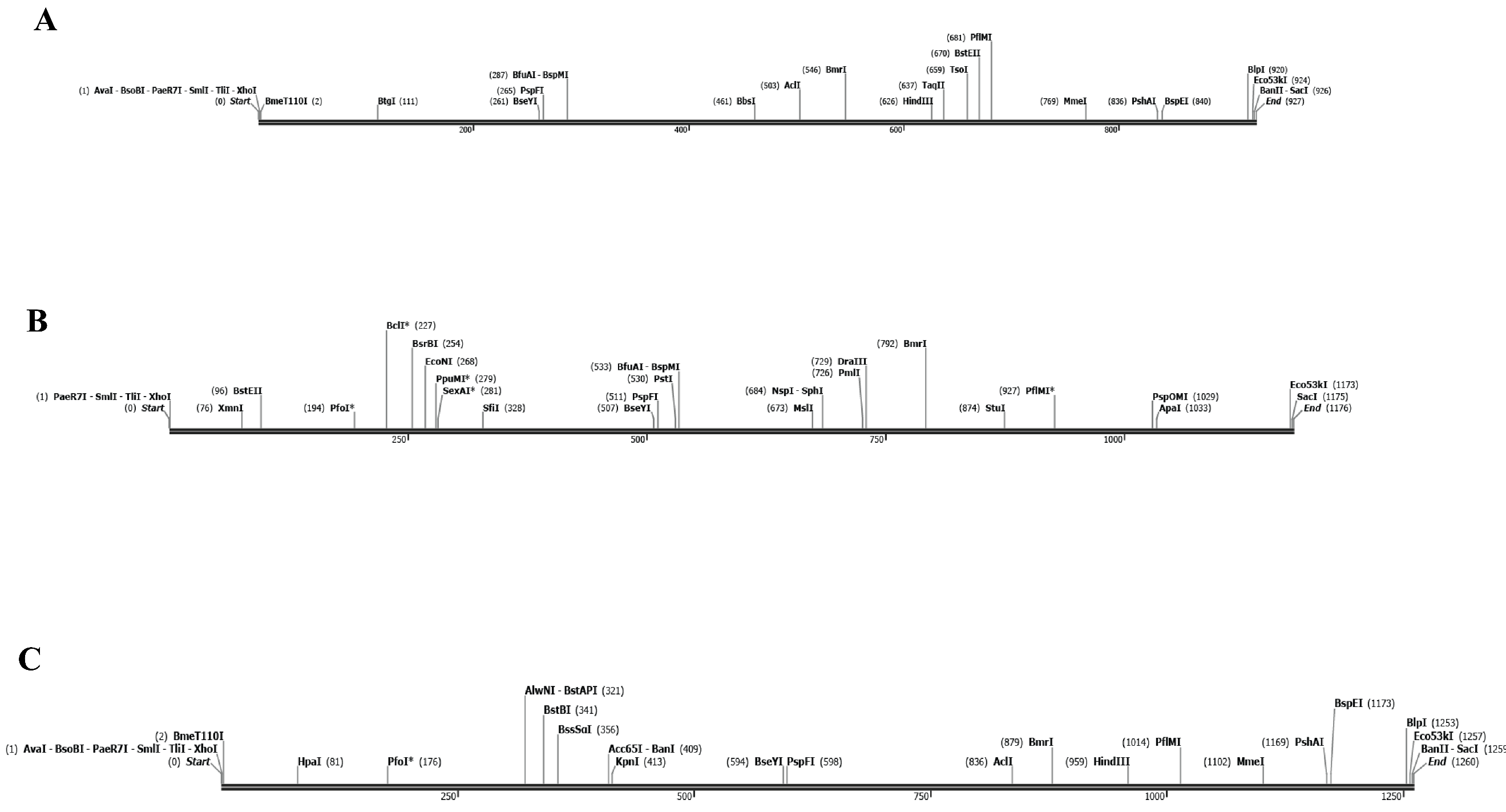


**Figure S7.** Optimized cDNA construct of **(A)** MPXV-1 **(B)** MPXV-2 **(C)** MPXV-3 with restriction enzymes sites added used for in silico cloning in the expression vector.


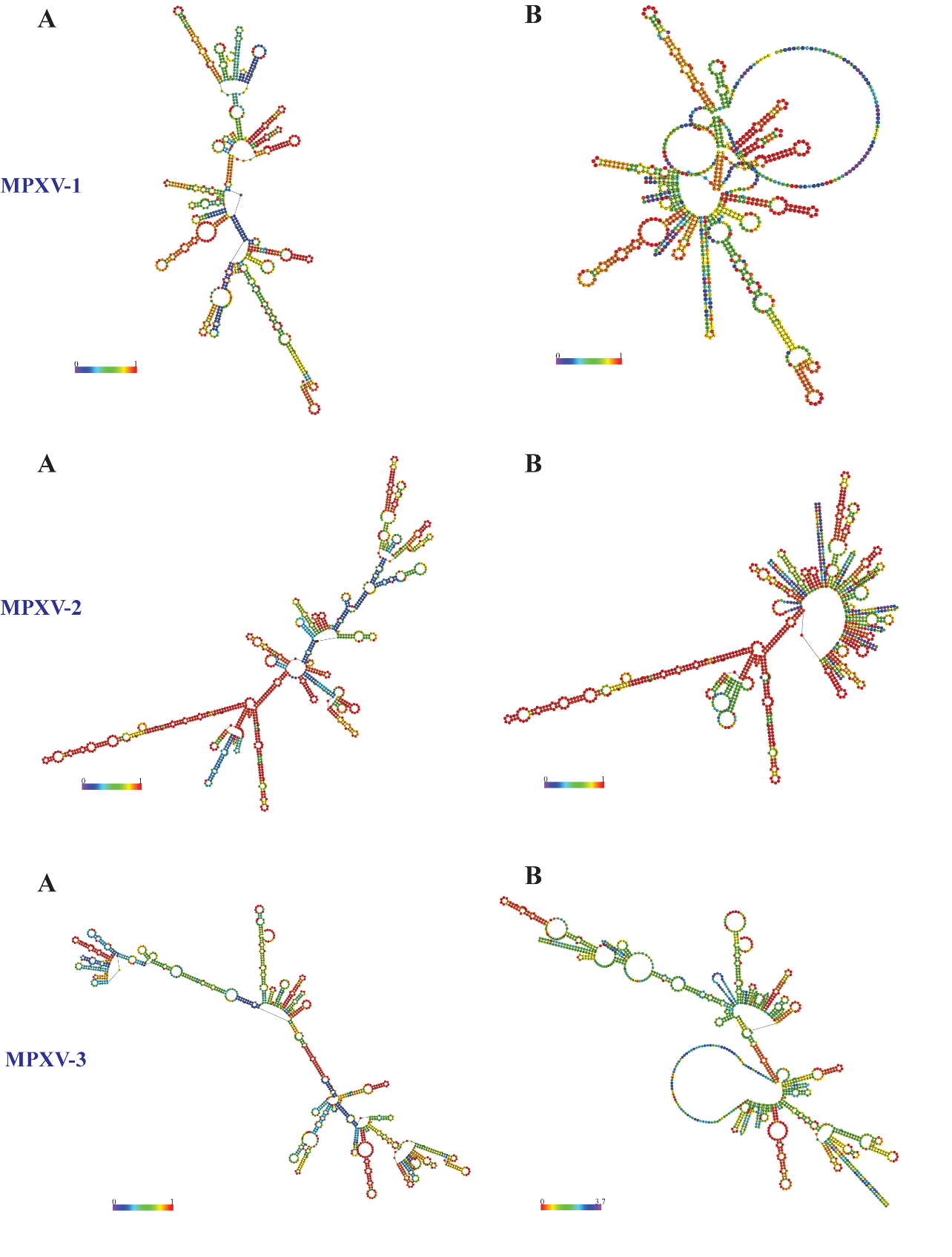


**Figure S8.** Predicted minimum free energy (MFE) mRNA secondary structure **(A)** and centroid mRNA secondary structure **(B)** of MPXV-1–3 (RNAfold program). The MFE structures are colored by base-pairing probabilities. For unpaired regions the color denotes the probability of being unpaired.


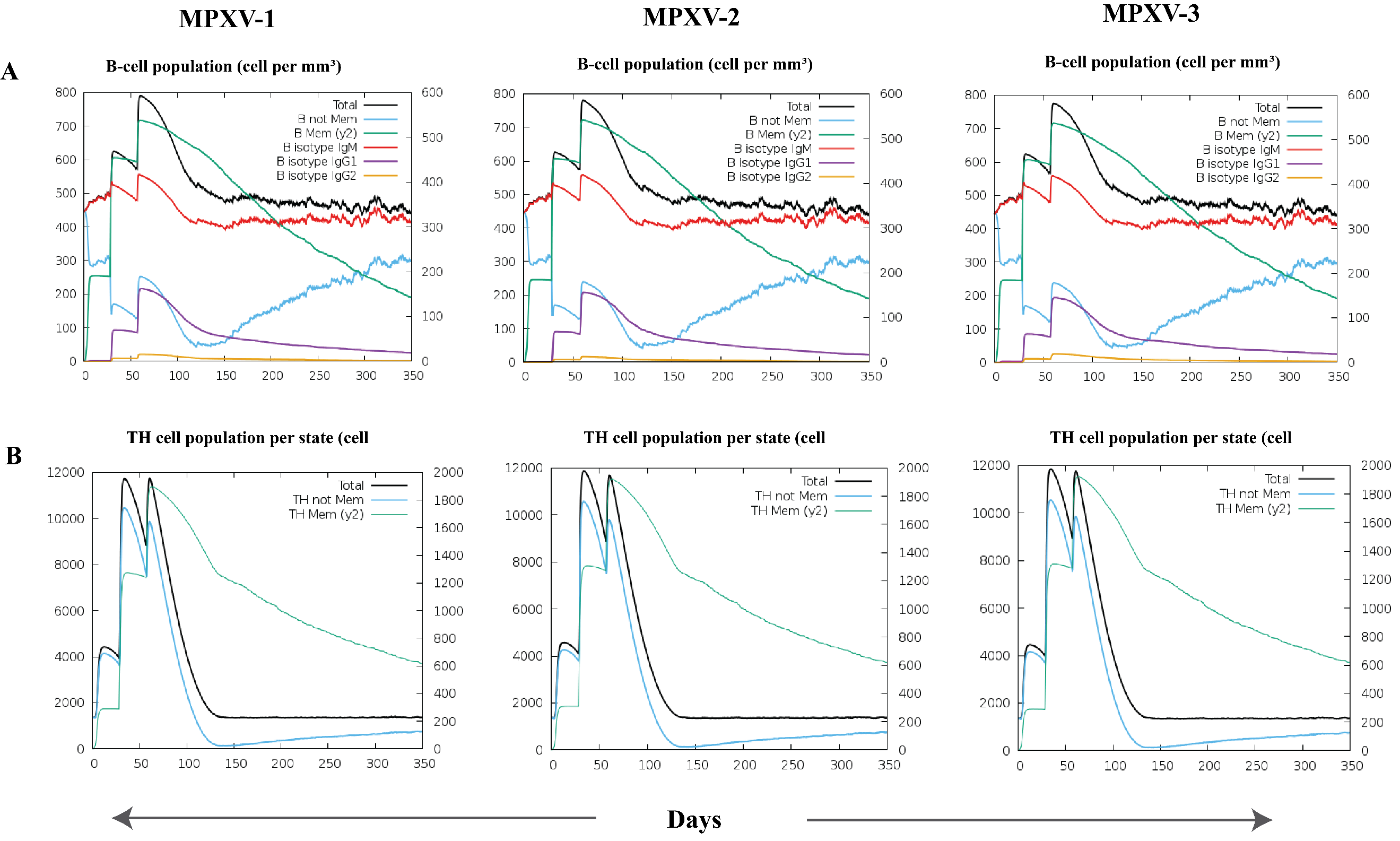


**Figure S9**. In silico immune simulation of an infection challenge, comprised of a virus responding to the sequence of MPXV proteins covered by the multi-epitope construct. **(A)** The total number of B-cell and isotypes per entity state. **(B)** The cell count of T-helper lymphocytes is shown in various forms, i.e., active, duplicating (in the mitotic cycle), resting (not active), and anergic.
